# NMDA and glycine receptors provide dual presynaptic regulation on climbing fibre inputs to cerebellar Purkinje cells

**DOI:** 10.1113/JP287456

**Published:** 2026-05-24

**Authors:** David C. H. Benton, Alan D. Robertson, Matthew Caldwell, Yuriy Pankratov, Lema Imam, Trevor G. Smart, Gareth Morris, Guy W. J. Moss

**Affiliations:** ^1^ Department of Neuroscience, Physiology and Pharmacology University College London London UK; ^2^ Division of Neuroscience, School of Biological Sciences, Faculty of Biology, Medicine and Health University of Manchester, Manchester Academic Health Science Centre Manchester UK

**Keywords:** climbing fibre, glycine, NMDA, presynaptic

## Abstract

**Abstract:**

Presynaptic receptors are important regulators of CNS activity. We have examined presynaptic receptor populations using a vibrodissociated rat cerebellar Purkinje cell preparation. In this preparation, NMDA receptor immunoreactivity co‐localized with antibody staining for the presynaptic marker synaptophysin. The application of NMDA increased the frequency of both inhibitory and excitatory synaptic currents, an effect that was blocked by the addition of 1 mM Mg^2+^. Similar stimulation of excitatory inputs occurred when applying a high concentration of glycine (100 µM) alone, a response that was blocked by 500 nm strychnine, but not by 1 mm Mg^2+^. Both glycine and NMDA stimulation produced many large amplitude excitatory postsynaptic currents of ∼200–600 pA. The size of these events suggests that they arise from climbing fibre, rather than parallel fibre, connections. In keeping with this idea, the largest events were consistently eliminated by tetrodotoxin (TTX) and isolated Purkinje neurons stained positively for the climbing fibre marker peripherin. Finally, we examined the effect of 100 µM glycine in a brain slice preparation. As was seen when using dissociated cells, a 100 µM concentration of glycine stimulated large postsynaptic currents and this effect was blocked by low concentrations of strychnine. Taken together, our findings indicate the presynaptic presence of NMDA and glycine receptors that can both operate as regulators of climbing fibre excitatory transmitter release.

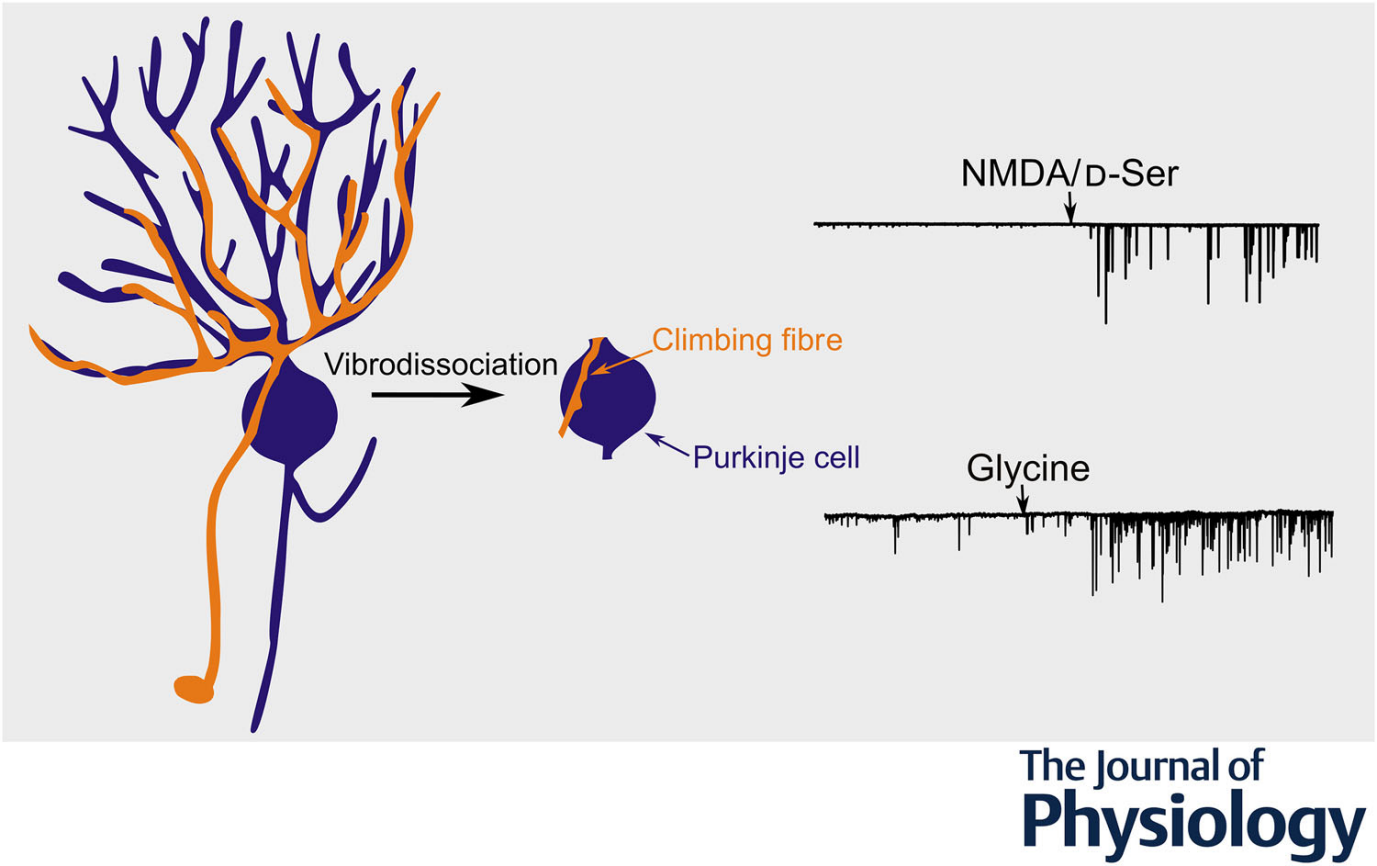

**Key points:**

- Functional presynaptic NMDA receptors are present on the cerebellar climbing fibre.
- Functional presynaptic glycine receptors are also present on the cerebellar climbing fibre.
- Both presynaptic NMDA and presynaptic glycine receptors can regulate the release of glutamate from the climbing fibre.
- In vibrodissociated neurons action potentials are generated locally to drive the largest climbing fibre events.

## Graphical abstract

**Abstract figure legend** Vibrodissociated Pukinje neurons retain active synaptic connections, including those from adhering fragments of the climbing fibre. These fragments generate excitatory synaptic events and the frequency of these events is increased by exposure to either NMDA [with d‐serine (d‐ser) as a co‐agonist] or glycine at a high (100 µM) concentration. These data imply a presynaptic modulatory role for both NMDA and glycine receptors expressed in the developing climbing fibre.

## Introduction

An increasingly large body of evidence shows that, in many regions of the CNS, NMDA and/or glycine receptors are located presynaptically where they play an important physiological role (e.g. Bouvier et al., 2015; Corlew et al., 2008; Duguid, 2013; Duguid & Sjöström, 2006; Duguid & Smart, 2009; Turecek & Trussell, 2001). In the cerebellum the first functional evidence for presynaptic NMDA receptors on Purkinje cell inputs was provided by Glitsch and Marty (1999). They reported that the application of NMDA increased the frequency of miniature inhibitory postsynaptic currents (mIPSCs), an effect that was inhibited by Mg^2+^. It has since been demonstrated that stimulating Purkinje neurons can lead to the retrograde release of glutamate (or a glutamate‐like transmitter), which acts on these presynaptic NMDA receptors and profoundly increases mIPSC frequency, a plasticity effect termed depolarization‐induced potentiation of inhibition (DPI) (Duguid & Smart, 2004). Via this mechanism, presynaptic NMDA receptors initiate a novel type of inhibitory synaptic plasticity and thus play an important functional role in setting the relative efficacy of inhibitory connections in the cerebellum.

Several studies have also suggested the presence of presynaptic NMDA receptors on excitatory terminals contacting Purkinje neurons. Early work by Petralia et al. (1994) indicated the presence of presynaptic NMDA receptors on parallel fibre terminals. Subsequently, experimental findings published by Casado et al. (2000, 2002) supported this idea and reported a functionally important role for presynaptic NMDA receptors on parallel fibre terminals. However, a later study by Shin and Linden (2005) suggested that the functional role initially ascribed to parallel fibre NMDA receptors might, instead, result from presynaptic NMDA receptors on interneuron terminals. Later, Bidoret et al. (2009) demonstrated the presence of presynaptic NR2A‐containing NMDA receptors at the cerebellar parallel fibre–Purkinje cell synapse, and suggested a functional role implementing a high‐pass filter synaptic plasticity rule. Finally, a later report by Lonchamp et al. (2012) showed that NMDA could increase the frequency of miniature excitatory postsynaptic currents (mEPSCs) recorded from Purkinje cells in young organotypic cultures. To date, however, presynaptic NMDA receptors have not been associated with excitatory climbing fibre–Purkinje cell connections.

Presynaptic glycine receptors have been found in a number of CNS regions including the brainstem (medial nucleus of the trapezoid body), spinal cord and hippocampus (e.g. Jeong et al., 2003; Kubota et al., 2010; Lee et al., 2009; Turecek & Trussell, 2001). Interestingly, these receptors have been identified as a potential therapeutic target for hyperekplexia (Xiong et al., 2014). For cerebellar Purkinje cells, Kawa (2003) made the first study of presynaptic glycine receptors reporting that at postnatal day (P)3–10, glycine increased the frequency of both IPSCs and EPSCs. However, in that study, only the IPSC effects were insensitive to tetrodotoxin (TTX). This was interpreted as meaning that EPSC regulation by glycine was occurring via a distant site capable of generating action potentials, most likely the granule cells.

To study the presynaptic receptors at cerebellar Purkinje cell synapses further, we started by returning to the nerve bouton preparation. In this preparation individual neurons, isolated by vibrodissociation, retain substantial numbers of active synaptic terminals, even though these terminals are structurally isolated from their axons and/or cell bodies (Akaike & Moorhouse, 2003; Vorobjev, 1991). We show that NMDA increases the frequency of inhibitory postsynaptic currents in this preparation. NMDA, co‐applied with either 10 µM glycine or d‐serine also increases the frequency of excitatory synaptic events, an effect that is blocked by MK801 or Mg^2+^, suggesting that the ionotropic action of NMDA receptors is essential for this response. Interestingly, at a higher concentration (100 µM), the application of glycine alone similarly increases the frequency of excitatory synaptic events. This latter effect is inhibited by co‐application of a low concentration of the glycine receptor antagonist strychnine (500 nM). Further, stimulation by either NMDA or a high glycine concentration increases the frequency of the largest amplitude excitatory events, which have peak currents of ∼200–600 pA. The application of TTX consistently blocks the biggest of these currents, indicating that they probably reflect action potential‐driven release from the climbing fibre. Immunostaining of the vibrodissociated cells shows that large climbing fibre fragments remain attached to the cell body. Finally, using a slice preparation we show that 100 µM glycine again stimulates large events, strikingly similar to those seen in the vibrodissociated preparation and that this effect is blocked by strychnine. Together, these observations suggest that the same presynaptic receptors can act to regulate climbing fibre release in both isolated and intact preparations and hence that new roles may emerge for these channels in the cerebellum.

## Materials and methods

### Ethical approval

All experiments were performed in accordance with UK Home Office regulations under the *Animals (Scientific Procedures) Act, 1986*. Sprague–Dawley rats (supplied by Charles River International, Wilmington, MA, USA) were maintained in a 12:12 h light/dark cycle and were given *ad libitum* access to food and water. P10 Sprague–Dawley rats of either sex were killed by cervical dislocation. The authors understand the ethical principles under which the Journal operates and confirm that this work complies with the Journal's animal ethics requirements.

### Vibrodissociated cell preparation and recording

Purkinje neurons were acutely dissociated from ∼500 µm thick parasagittal cerebellar slices. Once separated, brain slices were incubated in a continuously bubbled solution with 95% oxygen, 5% CO_2_ for at least 1 h, at room temperature (22–25°C) before being vibrodissociated. The incubation solution consisted of 135 mm NaCl, 3 mm KCl, 20 mm NaHCO_3_, 1 mm NaH_2_PO_4_, 15 mm glucose, 3 mm CaCl_2_ and 0.5 mm MgCl_2_ buffered with 95% O_2_ and 5% CO_2_. For vibrodissociation, slices were transferred to a solution of 145 mm NaCl, 3 mm KCl, 10 mm Hepes, 15 mm glucose, 0.5 mm CaCl_2_ and 3 mm MgCl_2_, pH adjusted to 7.35 with 1 m NaOH. The apparatus for vibrodissociation consisted of a 2.5 inch, 64 MΩ miniature loudspeaker (Farnell, Leeds, UK) mounted on a course manipulator with a glass probe attached to the loudspeaker cone. The probe was fashioned from a pipette pulled to a fine point at one end and fire polished to form a small glass ball. The loudspeaker was stimulated with a rectangular pulse, using a Grass S48 stimulator (Grass, Quincy, MA, USA) and the ball was moved slowly (vibrating at ∼80–130 Hz) just above the surface of the slice (∼30–100 µM). The slice was next removed from the dish and a period of approximately 10 min was allowed for cells to settle and adhere. The dish containing the cells was then transferred to the stage of an inverted microscope and perfused at a rate of 4–5 ml/min with an external solution composed of 145 mm NaCl, 3 mm KCl, 2.5 mm CaCl_2_, 10 mm Hepes and 10 mm glucose, pH adjusted to 7.3–7.35 with 1 m NaOH. Purkinje neurons were identified by their characteristic shape, thick dendritic stump and soma diameter (∼20–25 µm) and frequently also by the lack of a postsynaptic current in response to the application of NMDA. Patch pipettes were pulled from borosilicate glass (Harvard Biosciences, MA, USA) and had resistances of ∼2–5 MΩ when filled with internal solution (150 mM CsCl, 10 mM Hepes, 100 µM BAPTA or K_4_BAPTA, 1.5 mM MgCl_2_, 2 mM Na_2_ATP and 400 µM Na_2_GTP with the pH adjusted to 7.3 with 1 m CsOH). For many experiments this internal solution also contained 5 mM QX‐314 and 10 mM TEA chloride. All reagents were obtained from VWR with the exceptions of Hepes and CsCl (Calbiochem/Merck, Dorset, UK), NMDA, glycine, QX‐314, MK801 and TTX (Tocris, Bristol, UK), TEA chloride, 4‐AP (4‐aminopyridine), BAPTA, Na_2_ATP, Na_2_GTP, DNQX (6,7‐dinitroquinoxaline‐2,3‐dione), (+)‐bicuculline, strychnine and CsOH (Sigma‐Aldrich, St Louis, MO, USA) and K_4_BAPTA (Invitrogen, Paisley, UK).

Recordings were made using a HEKA EPC7 or EPC9 (HEKA, Lambrecht/Pfalz, Germany) amplifier with recordings digitised either via the EPC9 or by using a 1320A Digidata and pClamp 8 software (Molecular Devices, Sunnyvale, CA, USA). Recordings were sampled at 10 kHz and filtered at 2 kHz via a low‐pass Bessel filter. Cells were voltage‐clamped at −80 mV and recordings were made at room temperature (22–25°C).

### Slice recording methods

P10 Sprague–Dawley rats of either sex were obtained from the Biomedical Services Unit at University College London and killed by cervical dislocation. Brains were quickly dissected and submerged into ice cold and oxygenated sucrose artificial CSF (205 mM sucrose, 10 mM glucose, 26 mM NaHCO_3_, 1.2 mM NaH_2_PO_4_.H_2_O, 2.5 mM KCl, 5 mM MgCl_2_, 0.1 mM CaCl_2_). Brains were hemisected and mounted onto a slicing stage in sagittal orientation. Then, 300 µm thick cerebellar slices were obtained using a 7000 smz mk 2 vibrotome (Campden Instruments, Loughborough, UK) and stored submerged in oxygenated artificial CSF (125 mM NaCl,10 mM glucose, 26 mM NaHCO_3_, 1.25 mM NaH_2_PO_4_.H_2_O, 3 mM KCl, 1 mM MgCl_2_, 2 mM CaCl_2_) at room temperature. Slices were allowed to recover for at least 1 h and then transferred to a membrane chamber (Morris et al., 2016) for patch clamp recording, continuously perfused with oxygenated artificial CSF at room temperature and at ∼16 ml/min. Purkinje neurons were visually targeted and held in voltage clamp mode at −65 mV using an intracellular pipette solution composed of: 135 mM potassium gluconate, 4 mM KCl, 10 mM Hepes, 4 mM Mg‐ATP, 0.3 mM Na‐GTP and 10 mM Na_2_‐phosphocreatine; pH 7.3. Neurons were held in this configuration for at least 2 min before recordings. We then recorded baseline spontaneous EPSCs (sEPCSs) for at least 5 min, prior to application of 100 µM glycine (Sigma Aldrich/Merck, Dorset, UK). In a subset of neurons, we additionally applied 500 nm strychnine hydrochloride (Sigma Aldrich/Merck, Dorset, UK). We recorded one neuron from each brain slice. Recordings were rejected if access resistance exceeded 20 MΩ. Recordings were made using a CV‐7B headstage and Multiclamp 700B amplifier (both Molecular Devices) with 2 kHz Bessel filter, digitized at 20 kHz with a Power 1401 data acquisition interface (Cambridge Electronic Design, Cambridge, UK) and acquired using Spike2 v9 software (Cambridge Electronic Design).

### Experimental design and statistical analysis

Events in brain slices were detected using a semi‐automated MATLAB script and verified by the experimenter to reject false positives, as described by Morris et al. (2018). The amplitudes and cumulative distributions of slice sEPSC were analysed and generated using MATLAB (R2022a). For vibrodissociated cells events were identified using either Mini Analysis (Synaptosoft, Decatur, GA, USA) or in‐house software and the intervals between events were fitted as a mono‐exponential or, alternatively, a bi‐exponential distribution. Estimates of the exponential time constants and associated areas, along with the associated errors, were made directly from event intervals (not from the binned distributions) using the method of maximum likelihood (Colquhoun & Sigworth, 1995). This analysis was performed using a program written in the R environment for statistical computing. The weighted time constant for intervals,* τ_i_*_w_, was calculated using the formula:

$$\tau_{iw}=\frac{A_{1}\tau_{1}+A_{2}\tau_{2}}{\left(A_{1}+A_{2}\right)},$$

where *A*_1_ and *A*_2_ are the areas associated with time constants τ_1_ and τ_2_, respectively. The variance in *τ_i_*_w_ (σ^2^_τ_ *_i_*_w_) was then used to calculate the uncertainty in *τ_i_*_w_ via the relation:

$$\sqrt{\sigma_{\tau iw}^{2}}=\sqrt{\begin{array}{c} {\left(\frac{\partial \tau_{w}}{\partial \tau_{1}}\right)}^{2}\sigma_{\tau 1}^{2}+{\left(\frac{\partial \tau_{w}}{\partial \tau_{2}}\right)}^{2}\sigma_{\tau 2}^{2}+{\left(\frac{\partial \tau_{w}}{\partial A_{1}}\right)}^{2}\sigma_{A1}^{2}+{\left(\frac{\partial \tau_{w}}{\partial A_{2}}\right)}^{2}\sigma_{A2}^{2}+2\left(\frac{\partial \tau_{w}}{\partial \tau_{1}}\right)\left(\frac{\partial \tau_{w}}{\partial \tau_{2}}\right)\sigma_{\tau 1\tau 2}^{2}+2\left(\frac{\partial \tau_{w}}{\partial \tau_{1}}\right)\left(\frac{\partial \tau_{w}}{\partial A_{1}}\right)\sigma_{\tau 1A1}^{2}\\ +2\left(\frac{\partial \tau_{w}}{\partial \tau_{1}}\right)\left(\frac{\partial \tau_{w}}{\partial A_{2}}\right)\sigma_{\tau 1A2}^{2}+2\left(\frac{\partial \tau_{w}}{\partial \tau_{2}}\right)\left(\frac{\partial \tau_{w}}{\partial A_{1}}\right)\sigma_{\tau 2A1}^{2}+2\left(\frac{\partial \tau_{w}}{\partial \tau_{2}}\right)\left(\frac{\partial \tau_{w}}{\partial A_{2}}\right)\sigma_{\tau 2A2}^{2}+2\left(\frac{\partial \tau_{w}}{\partial A_{1}}\right)\left(\frac{\partial \tau_{w}}{\partial A_{2}}\right)\sigma_{A1A2}^{2} \end{array}}$$

where terms such as $\sigma_{\tau 1\tau 2}^{2}$ refer to the variance or covariance associated with the subscripted components (in this example the covariance of time constants τ_1_ and τ_2_). This relation was derived using standard rules of error propagation (see e.g. Salter, 2000). For display purposes, binned data were plotted using the transformation first suggested by Sigworth and Sine (1987).

Differences between weighted time constants were examined using a standard Student's *t* test, or via permutation tests, while differences in cumulative amplitude histograms were tested for using the Kolmogorov–Smirnov non‐parametric test (KS test) in R.

#### Immunohistochemistry

Purkinje cells, isolated by vibrodissociation as described above, were fixed in phosphate buffered saline (PBS; 136.9 mM NaCl, 2.7 mM KCl, 9.2 mM Na_2_PO_4_, 1.8 mM KH_2_PO_4_, pH 7.2) containing 40 mg/ml paraformaldehyde (Sigma), for 10 min at room temperature. The fixing solution was then removed, and the cells were rehydrated for 5 min in PBS, quenched for 10 min in PBS containing 50 mM ammonium chloride (Sigma) and washed again in PBS for 5 min. The fixed cells were then incubated for 1 h in antibody blocking solution (ABS; PBS 89.9%, fetal bovine serum (Thermofisher, Waltham, MA, USA) 10%, Triton‐X (Sigma) 0.1%, bovine serum albumin (Sigma, 5 mg/ml)) to permeabilize. Primary antibodies were suspended in ABS and were made to synaptophysin (dilution 1:200 dilution Novocastra), peripherin (dilution 1:100 dilution, Abcam, Cambridge, MA, USA), calbindin (dilution 1:1500 dilution, Millipore, Billerica, MA, USA) and GluN1 (dilution 1:1000 BD Pharmingen). Cells were then incubated in this mixture for 1 h before being washed three times in 1% Tween‐20 (Promega, Madison, WI, USA) and then incubated in secondary antibodies, suspended in ABS, for 1 h. Cells were then washed again three times in 1% Tween‐20 and stored in PBS overnight at 4°C and protected from light prior to imaging. Image analysis was performed using ImageJ and associated plugins.

#### FM‐143 staining of synaptic vesicles

Vibrodissociated Purkinje cells were incubated in a K^+^‐rich (50 mM) solution containing FM‐143 (Invitrogen, 5 µM) for 1–2 min. d‐AP5 (Sigma, 50 µM) and DNQX (5 µM) were included in the incubation solution to reduce excitotoxicity during depolarization. The solution was then replaced by standard bath solution containing FM‐143 and incubated for a further 8–10 min to allow internalization of the dye. Finally, the cells were washed three times in standard bath solution containing Advasep‐7 (Sigma, 1 mM) to quench dye that had not internalized. On some occasions, loading with FM‐143 was achieved by simply adding it to the cells and relying on spontaneous synaptic activity.

## Results

### Vibrodissociated Purkinje neurons retain both excitatory and inhibitory terminals

**Figure 1:**
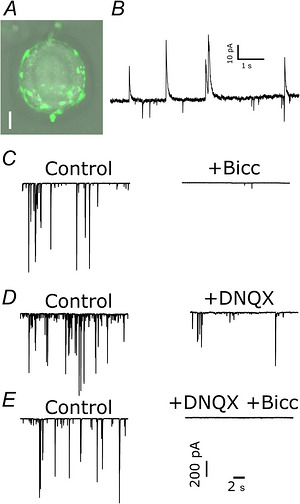
Vibrodissociated Purkinje cells retain active excitatory and inhibitory inputs *A*, a typical isolated Purkinje cell with a number of active boutons identified by FM‐143 loading (green dye). This image is a 3D confocal stack. Scale bar is 5 µm. A thick dendritic stump can be seen protruding from the top of the cell. *B*, whole cell recording at a holding potential of 0 mV. Events are clearly seen to include both inward and outward currents. Fast inward currents probably arise from excitatory synaptic events while the slower kinetics of outward currents are characteristic of inhibitory currents. *C*, whole cell recording at −80 mV in control conditions (left‐hand trace) and following the application of bicuculline (50 µM, right‐hand trace). Some events remain after blocking GABA_A_ receptors. *D*, whole cell recording before (left‐hand trace) and after (right‐hand trace) the application of DNQX (20 µM). Some events remain after blocking glutamate receptors. *E*, whole cell recording at −80 mV demonstrating that all events vanish when a combination of 20 µM DNQX and 50 µM bicuculline is applied. Similar responses were seen in eight out of eight cells (from eight slices and seven animals). Scale bars in the bottom right‐hand panel apply to all traces in *C*–*E*.

Vibrodissociation from P10 cerebellar slices produced isolated Purkinje neurons. An advantage of using rat Purkinje cells at this developmental stage is that they do not express NMDA receptors on the cell body (Farrant & Cull‐Candy, 1991; Monyer et al., 1994; Perkel et al., 1990). We were thus able to confirm that we had identified Purkinje neurons correctly by size, shape and a thick dendritic stump (Fig. 1*A*) and/or by checking for the absence of a whole cell current when NMDA was applied. Recordings from these isolated neurons revealed spontaneous synaptic currents of varying amplitude. Further, punctate staining was evident when the styryl dye FM1‐43 was loaded either spontaneously, or by active depolarization (with 50 mM KCl), consistent with the idea that these cells retain active terminals undergoing vesicle release and recycling (Fig. 1*A*).

We began our study by examining the nature of the spontaneous synaptic events we observed. At appropriate potentials (at, or close to, 0 mV under our recording conditions), and in the absence of any inhibitors, these synaptic events often ‘split’, producing both outward and inward currents (Fig. 1*B*). In these recordings the outward currents represented a subset of events with slow decay kinetics, characteristic of inhibitory inputs, whilst the inward currents had faster kinetics, characteristic of excitatory events. We were able to further confirm that there were mixed event types by first adding the selective GABA_A_ receptor antagonist bicuculline. The effect of bicuculline was to substantially reduce the event frequency, consistent with the idea that most events are mediated by GABA_A_ receptors (Fig. 1*C*). Similarly, when the selective AMPA receptor blocker DNQX was added, the frequency of events was also reduced (Fig. 1*D*), although the reduction was less dramatic than that seen with bicuculline. Finally, when DNQX was co‐applied with bicuculline, all synaptic currents were eliminated (Fig. 1*E*). Thus, in this preparation, whilst most spontaneous synaptic activity is inhibitory in nature, both excitatory and inhibitory synaptic terminals can remain functionally attached to these isolated cells.

### Mg^2+^‐sensitive presynaptic NMDA receptors modify transmitter release in vibrodissociated Purkinje neurons

**Figure 2:**
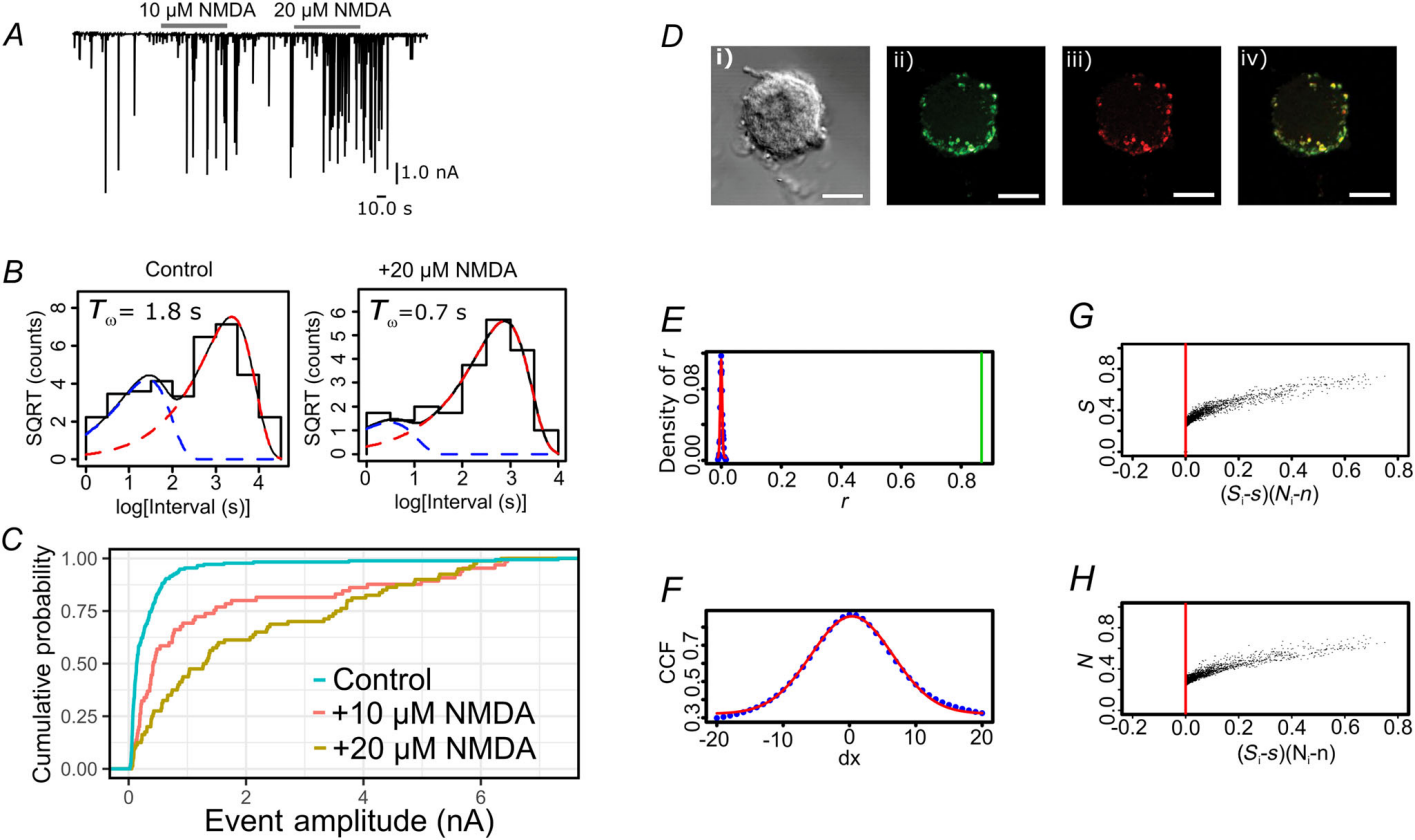
Presynaptic NMDA receptors are present and active in the Purkinje cell nerve bouton preparation *A*, a continuous recording from an isolated Purkinje cell showing increased synaptic event frequency following the application of NMDA, first at 10 µM, then at 20 µM, each time applied with 10 µM glycine as a co‐agonist. Similar responses were seen in six out of seven cells (from seven slices and five animals). *B*, the distribution of intervals between synaptic events was usually best fit by the sum (continuous line) of two exponentials (dotted lines in red and blue). Hence, we calculated a weighted time constant (*τ_i_*_w_) to characterize inter‐event intervals. Increases in event frequency (decreases in *τ_i_*_w_) are seen when the control period (upper left‐hand panel; *τ_i_*_w_ = 1.8 ± 0.2 s) is compared with the period of 20 µM NMDA application (lower left‐hand panel *τ_i_*_w_ = 0.7 ± 0.1 s). *C*, NMDA application affects the distribution of event amplitudes creating an increase in the proportion of large amplitude events (KS test *P* < 0.001 for control *vs*. 10 µM NMDA and for 10 µM NMDA *vs*. 20 µM NMDA). *D*, (i–iii) brightfield and fluorescence confocal images of a representative Purkinje cell showing immunoreactivity for synaptophysin (green) and the NMDA receptor subunit GluN1 (red). *D*iv shows colocalization (yellow) of synaptophysin and GluN1 markers. Scale bars: 10 µm. Images are composites of several stack slices but subsequent analysis was performed on single confocal slices. Colocalization statistics for this cell were calculated using JACoP. *E*, Costes’ randomization test assessing Pearson correlation coefficient ‘*r*’ (1000 randomizations, actual correlation *r* = 0.868, *P* < 0.001); *F*, Van Steensel's cross‐correlation function (CCF), with strong central peak; *G* and *H*, Li's intensity correlation plots. Pixel intensities for synaptophysin (S) and GluN1 (N) with means *s* and *n*, respectively, show characteristic curvature in the positive quadrant (ICQ = 0.432).

We next examined the effects of NMDA (with 10 µM glycine as a co‐agonist) on both synaptic current amplitudes and frequencies in a mixed population of inhibitory and excitatory events. As illustrated in Fig. 2*A*, NMDA typically produced a robust frequency increase (reduced mean inter‐event interval), a hallmark of presynaptic regulation. In analysing the frequency of these spontaneous events, stochastic bursting behaviour was often seen both before and during NMDA application. Thus, the distribution of inter‐event intervals was often best fit by the sum of two exponentials (Fig. 2*B*). We have not investigated the basis of this behaviour. However, to assess changes in event frequency due to presynaptic receptor responses, we calculated a weighted average of the two fitted time constants (*τ_i_*_w_) to provide an appropriate measure of mean inter‐event intervals.

The increased frequency of events produced by NMDA was accompanied by an increase in the proportion of synaptic currents with large amplitudes (Fig. 2*C*). For some cells, a concentration of 10 µM NMDA was sufficient to produce large increases in event frequency. In other cells, higher concentrations of 20 µM NMDA, or even 50 µM, were required to produce similar responses. However, at the highest concentration of NMDA (50 µM), synaptic events tended to run down, so in many experiments we used no more than 20 µM as the test NMDA concentration. At this concentration, we observed NMDA‐induced frequency increases in the mixed event population for six out of seven cells examined (from seven slices and five animals).

The increased frequency of synaptic events in response to NMDA suggests a presynaptic location for these receptors. To confirm this, we used NMDA receptor antibodies to see if receptor immunoreactivity would co‐localize with synaptic markers. We thus stained vibrodissociated neurons with antibodies to both the obligate NMDA receptor subunit GluN1 and the presynaptic protein synaptophysin. Consistent with a presynaptic location, antibody staining for synaptophysin highlighted small punctate regions located around the cell's perimeter (Fig. 2*D*). Further, immunoreactivity for the GluN1 subunit co‐localized strongly with the immunoreactive sites for synaptophysin (Fig. 2*D*–*H*). All cells examined (*n* = 10 cells, one animal) showed a strong positive colocalization of immunoreactivity for GluN1 and synaptophysin. The median Pearson correlation coefficient was 0.839 (Costes randomization *P* < 0.001 for all cells), whilst the median intensity correlation quotient (ICQ) was 0.418. The finding of such strong colocalization suggests the NMDA receptors that are present in the Purkinje cell nerve–bouton preparation are expressed at synapses. This, along with the lack of postsynaptic currents mediated by NMDA receptors, accords with the idea that they function presynaptically and thus agrees with several earlier reports in the literature (e.g. Duguid & Smart 2004).

**Figure 3:**
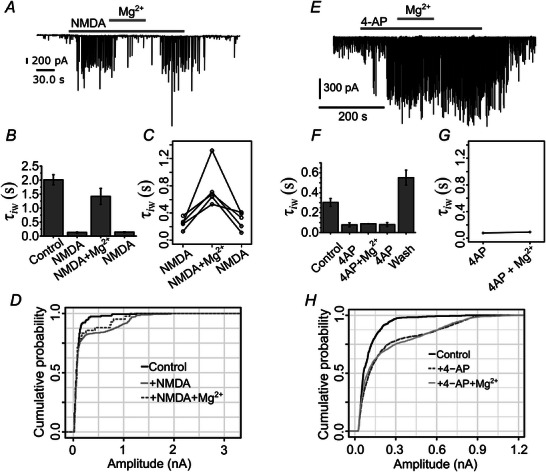
Mg^2+^‐sensitive NMDA receptor responses mediate increased event frequencies *A*, Purkinje cell recording showing that the NMDA‐driven increase in event frequency is blocked by Mg^2+^ (1 mM). *B*, mean event interval (*τ_i_*_w_) calculated for the record shown in *A* during the control period, the first period of NMDA (20 µM) application, then NMDA co‐applied with 1 mM Mg^2+^, and second period of NMDA only (Mg^2+^ wash). *C*, changes in mean inter‐event intervals for five neurons before, during and after Mg^2+^ is applied in addition to NMDA (five cells, five slices, three animals). The application of Mg^2+^ reduced the frequency of synaptic events (*P* < 0.001). *D*, the application of NMDA and glycine increases the proportion of events with a large amplitude (*P* = 0.0155, KS test for the record in *A*). *E*, Purkinje cell recording showing that a similar increase in event frequency driven by 4‐AP (50 µM) is only slightly affected (<20%) by 1 mM Mg^2+^. *F*, mean interval between events for the record shown in *E* during control, 4‐AP application, 4‐AP and Mg^2+^, return to 4‐AP only, and return to control (wash) shown left to right. *G*, data from two paired recordings showing only very small changes in inter‐event interval following the application of 1 mM Mg^2+^ when release is stimulated by 4‐AP. Data are shown on the same scale used in *C* for comparison. *H*, cumulative plot for the trace shown in *E*. Mg^2+^ at 1 mM co‐applied with 4‐AP causes a small change in the distribution of amplitudes, but this is minimal compared to the change produced by 4‐AP alone (*P* < 0.001, control *vs*. 4‐AP, KS test). Bar graph values in *B* and *F* are given as the mean ± SD of the mean.

Many, but not all, NMDA receptor responses are sensitive to block by Mg^2+^. For example, some responses occur via a metabotropic pathway that does not require ion conduction (see e.g. Bouvier et al., 2018). Indeed, responses from presynaptic NMDA receptors have been reported with Mg^2+^ present (Duguid & Smart, 2004; Shin & Linden, 2005). Therefore, we examined the Mg^2+^ sensitivity of NMDA‐mediated responses by first applying 20 µM NMDA and then co‐applying 1 mM Mg^2+^ with NMDA, before finally returning to a Mg^2+^‐free NMDA solution. In these experiments Mg^2+^ substantially and reversibly blocked the NMDA effect, increasing the mean interval between events when compared with NMDA alone (*n* = 5 cells, five slices from three animals, *P* < 0.001, Fig. 3*A*–*C*). As shown in Figs 2 and 3*D*, typically the proportion of large amplitude events increases following the application of NMDA. With the subsequent application of 1 mM Mg^2+^ some large events remain, possibly so‐called maximinis (Llano et al., 2000), but the frequency of large amplitude events is greatly reduced. These observations are consistent with the stimulation in event frequency being mediated by an Mg^2+^‐sensitive NMDA receptor response. However, to determine if Mg^2+^ might be affecting neurotransmitter release probability by some other route that does not involve NMDA receptors, we examined the effect of 1 mM Mg^2+^ on a similar event frequency increase induced by 50 µM 4‐AP. The application of 4‐AP dramatically increased event frequency and also increased the proportion of large amplitude events but when Mg^2+^ was co‐applied with 4‐AP, in the absence of NMDA, there was only a small reduction in event frequency (19% increase in mean inter‐event interval) and no significant change in the distribution of event amplitudes (Fig. 3*E*–*H*). Thus, 1 mM Mg^2+^ does not have a major effect on event frequency in the absence of NMDA receptor stimulation. Instead, these findings suggest that NMDA is affecting release via Mg^2+^‐sensitive NMDA receptors and an ionotropic mechanism.

### Presynaptic NMDA receptors regulate both inhibitory and excitatory connections

**Figure 4:**
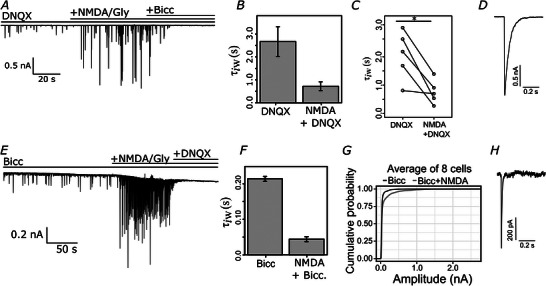
NMDA, co‐applied with a low (10 µM) concentration of glycine, increases the frequency of both inhibitory and excitatory events *A*, a recording from an isolated Purkinje cell in the presence of DNQX (20 µM). *B*, co‐application of NMDA (50 µM) reduced inter‐event intervals shown for the record in *A*. *C*, pooled data showing the decrease in inter‐event interval for five cells (from five slices and five animals) following the application of NMDA (*P* = 0.0231). All events are blocked when bicuculline is co‐applied (*A*). Characteristic events in the presence of DNQX have slow kinetics, typical of GABAergic transmission (*D*). *D*, a recording from an isolated Purkinje neuron in the presence of bicuculline (50 µM). The subsequent addition of NMDA (50 µM) with glycine (10 µM) increases event frequency but these events are blocked once both bicuculline and DNQX are added. *F*, inter‐event interval before and after the application of NMDA for the record shown in *E*. Error bars in *F* and *B* represent the standard deviation of the mean. *G*, there is also an increase in the proportion of larger amplitude events (KS test, *P* < 0.001, *n* = 8 cells, eight slices, five animals). *H*, these events have faster kinetics characteristic of glutamatergic transmission.

To examine the types of synaptic inputs being regulated by NMDA, we began by applying NMDA (with 10 µM glycine as a co‐agonist) along with DNQX (20 µM), circumstances under which only inhibitory synaptic currents remain unblocked. Co‐application of NMDA in the presence of DNQX substantially increased the frequency of synaptic currents (*n* = 5 cells from five slices and five animals; Fig. 4*A*–*C*). These events vanished when bicuculline was subsequently perfused in addition to DNQX (Fig. 4*A*), and they show a slow kinetic behaviour (Fig. 4*D*), thus confirming that NMDA was acting to increase the frequency of inhibitory synaptic currents. These observations thus support several previous studies indicating the presence of presynaptic NMDA receptors on interneuron axon terminals (e.g. Duguid & Smart, 2004; Glitsch & Marty, 1999; Shin & Linden, 2005).

**Figure 5:**
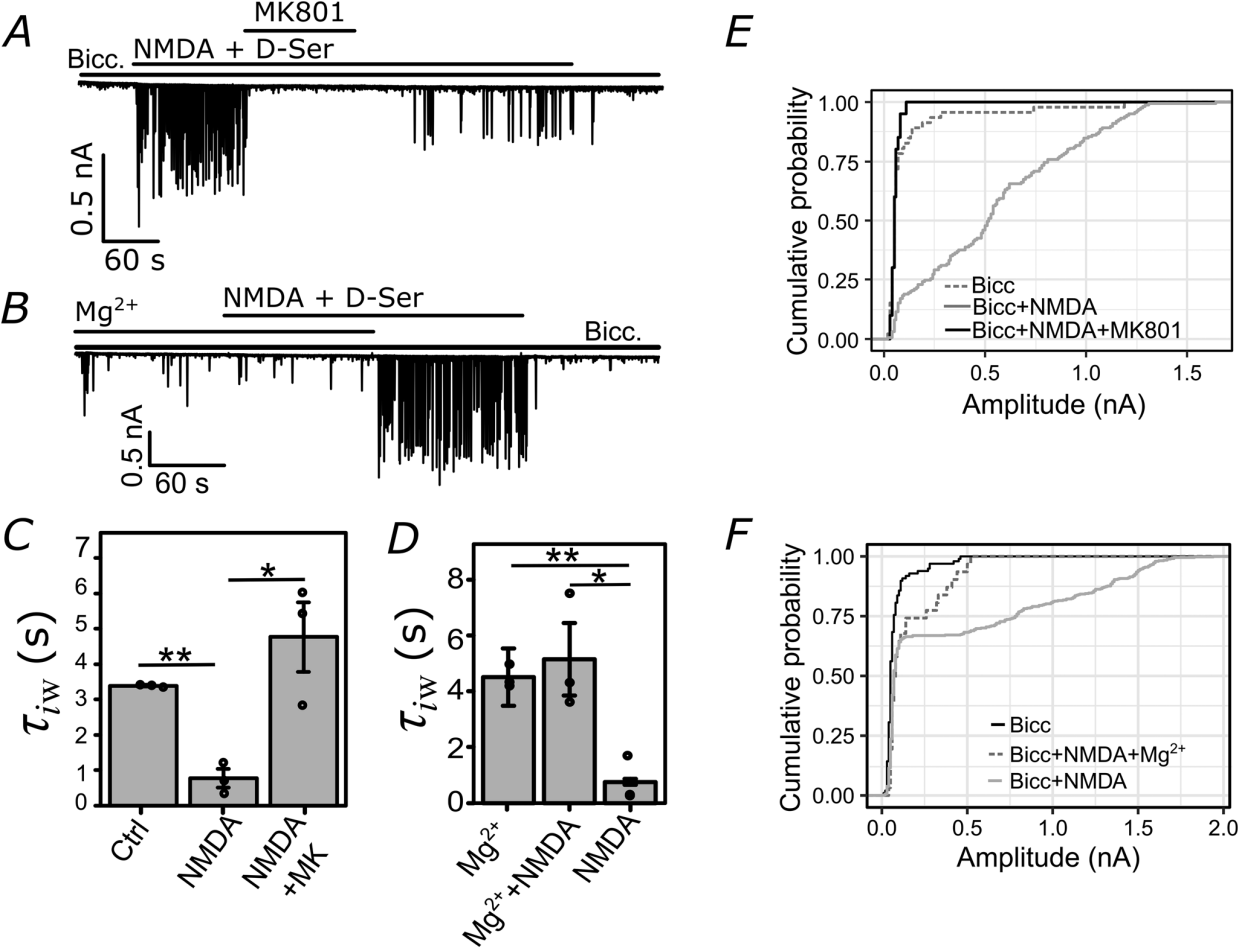
NMDA increases event frequency with d‐serine as the co‐agonist *A*, Purkinje cell recording in the presence of bicuculline, showing the effect of adding NMDA (50 µM) with d‐serine (10 µM) as a co‐agonist. The up‐regulation of event frequency is blocked by the application of MK801 (10 µM), which washes out slowly because it is slow to dissociate from the NMDA receptors. *B*, Mg^2+^ (1 mM) also blocks the NMDA‐driven event frequency increase that is seen when NMDA and d‐serine are co‐applied (*n* = 3 cells, three slices, two animals) in the presence of bicuculline. *C*, changes in *τ_i_*_w_ values averaged over three experiments of the type shown in *A*. NMDA with d‐serine increased the event frequency (*P* = 0.0107) whilst MK801 reduced event frequency (*P* = 0.0492, *n* = 3 applications, two cells, two slices, one animal). *D*, changes in *τ_i_*_w_ values averaged over three experiments of the type shown in *B* (three cells, three slices, two animals). Removing Mg^2+^ in the presence of NMDA and d‐serine significantly increases event frequency (*P* = 0.0269 Mg^2+^ + NMDA *vs*. NMDA and *P* = 0.0041 Mg^2+^ *vs*. NMDA). The distribution of event amplitudes for traces shown in *A* and *B* are plotted cumulatively in *E* and *F*, respectively. Applying the KS test to the data in *E* gave *P* values of <0.001 (Bicc *vs*. Bicc/NMDA and Bicc/NMDA *vs*. Bicc/NMDA/MK801) and for the data in *F P* = 0.00877 (Bicc/NMDA/D‐Ser/Mg^2+^ *vs*. Bicc/NMDA/D‐Ser). Error bars represent the standard deviation of the sample means.

We next examined the effect of NMDA on excitatory events by co‐application with bicuculline to block inhibitory transmission. Application of NMDA (again with 10 µM glycine as a co‐agonist) substantially increased the frequency of synaptic events (Fig. 4*E*, *F*) and increased the proportion of larger events (eight cells, eight slices, five animals, *P* < 0.001, Fig. 4*G*). These events were blocked by the subsequent addition of DNQX and had fast kinetics (Fig. 4*E*, *H*), thus confirming that they arise from excitatory inputs. In performing these early experiments, we noted that in the absence of NMDA, 10 µM glycine sometimes increased event frequency by itself, although this effect was much smaller than that seen when NMDA was co‐applied. Consequently, to better isolate responses mediated by NMDA receptors we examined the activity of NMDA when d‐serine (10 µM) was used as the co‐agonist. We found that the combination of NMDA and d‐serine produced a similar up‐regulation of synaptic current frequency (*n* = 5 cells from five slices and three animals) to that seen when NMDA was applied with glycine as the co‐agonist (Fig 5*A*, *B*). Further, this effect of NMDA and d‐serine was blocked both by the application of MK801 (10 µM) and by 1 mM Mg^2+^ (Fig. 5*A*–*D*). Changes in the distribution of event amplitudes are shown in Fig. 5*E* and *F*. It is notable that co‐application of NMDA and serine increased the proportion of events with large amplitudes. Altogether, although the magnitude of NMDA up‐regulation was variable with respect to both the increase in frequency and to the change in distribution of amplitudes (compare Figs 4*G* and 5*F*), a frequency response was clearly evident in 39 of 48 cells taken from 48 slices and 21 different animals (using either glycine or serine as a co‐agonist). Thus, at this developmental stage it seems that Mg^2+^‐sensitive presynaptic NMDA receptors can regulate excitatory inputs to Purkinje cells.

### Glycine receptors also regulate release from excitatory terminals

**Figure 6:**
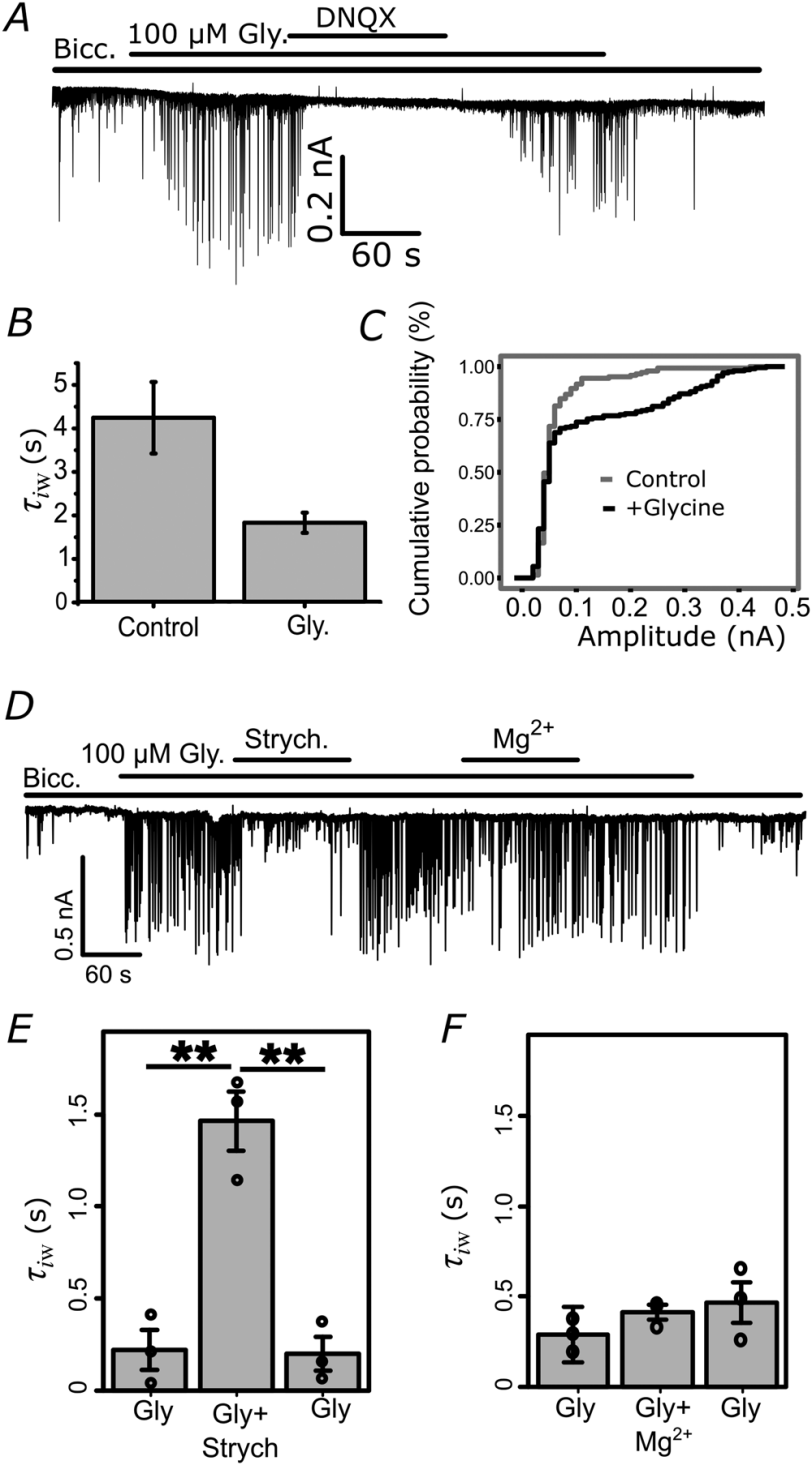
Glycine receptors regulate excitatory synaptic event frequency in vibrodissociated Purkinje cells *A*, recording from a vibrodissociated Purkinje cell in the presence of bicuculline (50 µM). The application of a high concentration of glycine (100 µM) increased the frequency of excitatory currents, including those with the largest amplitudes. All events are eliminated by the subsequent co‐application of DNQX (20 µM). Similar responses to glycine were seen in 13 of 14 recordings (14 cells from 14 slices and eight animals). *B*, the effect of 100 µM glycine on inter‐event intervals for the record shown in *A*, reflecting the event frequency increase. Error bars represent the standard deviation of the mean. *C*, cumulative distribution of sEPSC amplitudes in the absence and presence of glycine (100 µM) for the record shown in *A*. Glycine causes a marked increase in the proportion of large amplitude sEPSCs (KS test, *P* = 0.00208). *D*, whole cell recording illustrating the effect of strychnine (500 nm) and the absence of an effect of Mg^2+^ (1 mM) on sEPSCs stimulated by glycine (100 µM). *E*, inter‐event intervals showing the effect of strychnine on glycine‐stimulated event frequencies, (pooled data, *n* = 3 cells from three slices and two animals). Strychnine increases the inter‐event interval (*P* = 0.0078 before *vs*. during strychnine application and *P* = 0.0065 glycine and strychnine *vs*. glycine only wash). *F*, inter‐event intervals illustrate the lack of effect of 1 mM Mg^2+^ on glycine‐stimulated event frequencies (pooled data, *n* = 3 cells, from three slices and one animal, *P* = 0.412). In both *E* and *F* error bars represent the standard deviation of the mean.

At a high concentration (100 µM), glycine alone produced a profound increase in event frequency in 13 of 14 cells from eight animals, as illustrated in Fig. 6*A* and *B*. It also produced an increase in the proportion of events with larger amplitudes (Fig. 6*C*). These events, recorded in the presence of bicuculline, were blocked by the co‐application of DNQX, demonstrating that they arise from excitatory inputs. To determine whether these responses were mediated by glycine receptors (rather than NMDA receptors), we performed two additional tests. First, we examined the effect of applying 500 nm strychnine. At this concentration, strychnine remains selective for glycine receptors over GABA_A_ receptors (Jonas et al., 1998) although, given that it is a competitive antagonist, at this low concentration, it is not expected to completely abolish glycine receptor responses. In keeping with this idea, we found that 500 nM strychnine substantially reduced (*P* = 0.0078, pooled data, *n* = 3 cells from three slices and two animals), but did not completely eliminate, the increase in event frequency seen when 100 µM glycine was applied alone (Fig. 6*D*, *E*). As an additional test, we next examined the sensitivity of this glycine effect to the application of 1 mM Mg^2+^. If the effect were somehow mediated by NMDA receptors, it should be sensitive to Mg^2+^ block (Figs 3 and 5). We found that the increase in event frequency produced by 100 µM glycine alone was not sensitive to 1 mM Mg^2+^ (Fig. 6*D*, *F*; *n* = 3 cells from three slices and one animal, *P* = 0.412). These observations suggest that the increase in excitatory event frequency seen with high concentrations of glycine is mediated by presynaptic glycine receptors.

### Climbing fibre fragments remain attached to vibrodissociated neurons

**Figure 7:**
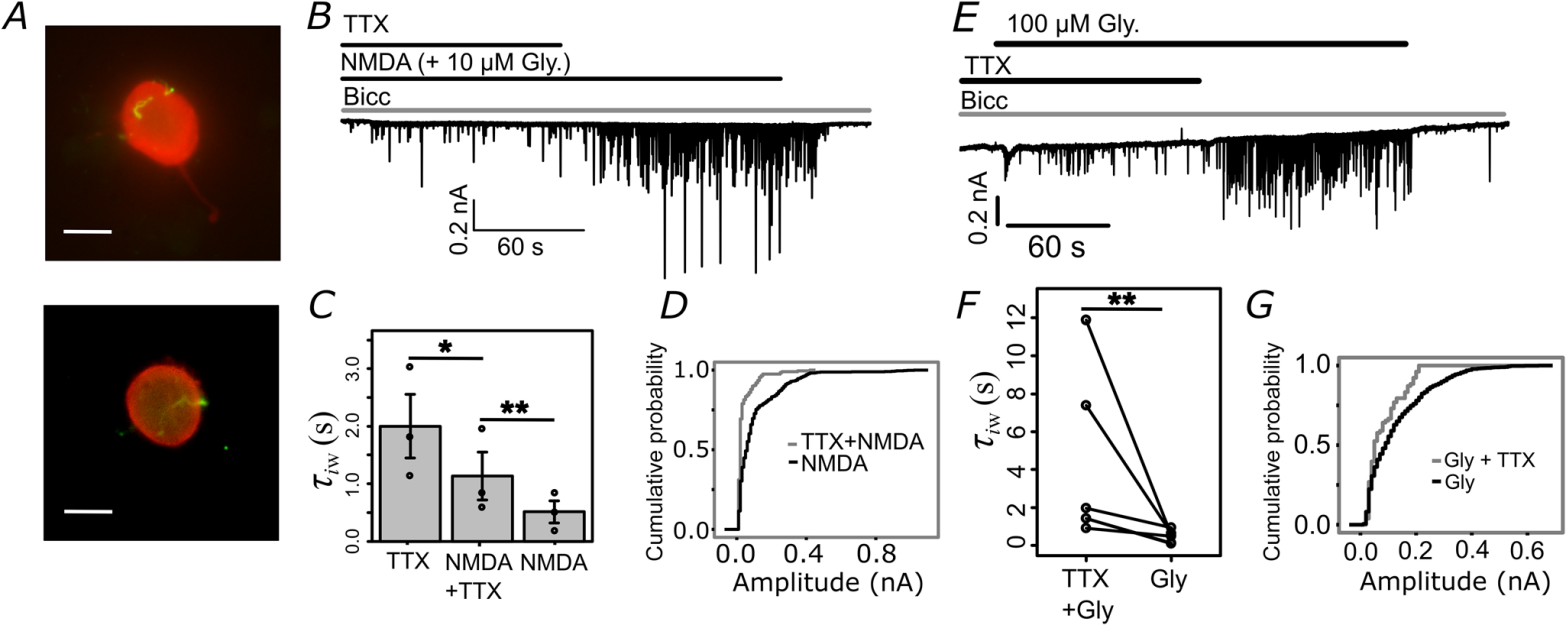
NMDA and glycine receptors drive the generation of action potentials in climbing fibre fragments to produce the largest event amplitudes and frequencies *A*, vibrodissociated neurons stained for calbindin (a Purkinje cell marker, red) and peripherin (green, a marker of the climbing fibre). Scale bar is 13 µm. *B*, a continuous recording from a vibrodissociated Purkinje cell in the presence of bicuculline to isolate excitatory events. TTX (100 nm) supresses the effect of NMDA on event frequency (*P* < 0.001) and eliminates the largest amplitude events. *C*, analysis of inter‐event intervals from three experiments of the type shown in *B* (three cells, three slices, two animals). An increase in event frequency is seen when NMDA is applied with TTX (*P* = 0.0500), and intervals get shorter again when TTX is removed (*P* < 0.001). *D*, cumulative distribution of amplitudes of NMDA‐stimulated sEPSCs in the absence and presence of TTX for the record in *B*. A clear increase (*P* < 0.001, KS test) in the fraction of large amplitude events is evident when TTX is absent. *E*, a Purkinje cell recording showing the impact on excitatory events when TTX (100 nm) and glycine (100 µM) are applied. TTX (100 nm) supresses the effect of glycine on event frequency (*P* = 0.0015) and eliminates the largest amplitude events. *F*, analysis of inter‐event intervals from four experiments of the type shown in *E* (*n* = 4 cells from four slices and three animals). The frequency of events increased when TTX was removed (*P* = 0.01435). *G*, cumulative distribution of amplitudes of glycine‐stimulated sEPSCs in the absence and presence of TTX for the record in *E*. An increase in the fraction of large amplitude events is evident when TTX is removed (*P* = 0.00476, KS test).

Purkinje neurons receive excitatory inputs from both parallel fibres (targeting the Purkinje cell dendritic field) and climbing fibres (that innervate both the Purkinje cell soma and dendrites), so, if a small dendritic stump is retained, excitatory synaptic currents could arise from either or both of these types of connections. Further, presynaptic NMDA receptors have been associated with parallel fibre terminals in the past (e.g. Casado et al., 2000) whilst no association has been reported for the climbing fibre. However, the amplitudes of the evoked excitatory events following both NMDA and glycine stimulation cover a considerable range that includes very large events, many of which are ∼200–600 pA (Figs 4, 5, 6) or greater. Single parallel fibres typically produce only small amplitude currents (most <30 pA at −70 mV) and are less likely to be present in our vibrodissociated preparation. Indeed, in a study by Isope and Barbour (2002), even granule cell axons ascending close to the Purkinje cell body failed to make powerful connections and only on a single occasion did they record a current greater than 100 pA (∼120 pA). Therefore, to generate the largest events that we see, parallel fibre terminals would have to be releasing transmitter in a highly coordinated fashion. Given the nature of our isolated cell preparation, this seems implausible. By contrast, the only other excitatory connection to Purkinje cells, the climbing fibre, is well known to generate large (nA) currents in the Purkinje cell body by a coordinated release of vesicles that is driven by action potentials (Konnerth et al., 1990). For this reason, we set out to determine whether fragments of the climbing fibre remain attached to the Purkinje cell body after vibrodissociation. To examine this possibility, we stained vibrodissociated neurons using an antibody to peripherin, an intermediate filament protein, and a selective marker for the climbing fibre (Errante et al., 1998). To identify the population of Purkinje cells, we co‐stained with calbindin, a specific marker for this cell type in the cerebellum (Bastianelli, 2003). We found that the calbindin‐positive Purkinje cells had thin, rope‐like strands of peripherin immunoreactivity associated with them, on and around the cell body (Fig. 7*A*). Thus, it seems that climbing fibre fragments, retained by the Purkinje cells after vibrodissociation, probably account for the large amplitude excitatory currents. That being the case, we then determined whether these large currents arise simply through depolarization of the climbing fibre, and activation of voltage‐gated calcium channels, or whether NMDA and glycine generate action potentials to drive release. To approach this question, we examined the impact of TTX on NMDA‐ and glycine‐stimulated synaptic activity.

### Action potentials generate the largest amplitude events stimulated by glycine and NMDA

We recorded the effect of applying 50 µM NMDA (with 10 µM glycine) to vibrodissociated Purkinje neurons either with or without TTX (100 nm) and in the continual presence of bicuculline (Fig. 7*B*). Whilst TTX was present, it curtailed the stimulatory impact of NMDA on both event frequency and amplitude (Fig. 7*B*–*D*, KS test for amplitude changes, *P* < 0.001). Of particular interest is that TTX seemed to completely prevent the occurrence of the very largest synaptic currents, suggesting that action potentials are necessary to generate these events. A similar picture was observed when a high concentration (100 µM) of glycine alone was used to stimulate release either in the absence or presence of TTX. Whilst the application of glycine still increased the event frequency in the presence of TTX (Fig. 7*E*, *F*), the largest amplitude events appeared only when TTX was removed, as did the highest event frequency (Fig. 7*E*–*G*). Given these results, we suggest that both NMDA and glycine receptors can regulate climbing fibre release in vibrodissociated rat Purkinje cells, and in vibrodissociated neurons they generate action potentials that create large amplitude currents.

**Figure 8:**
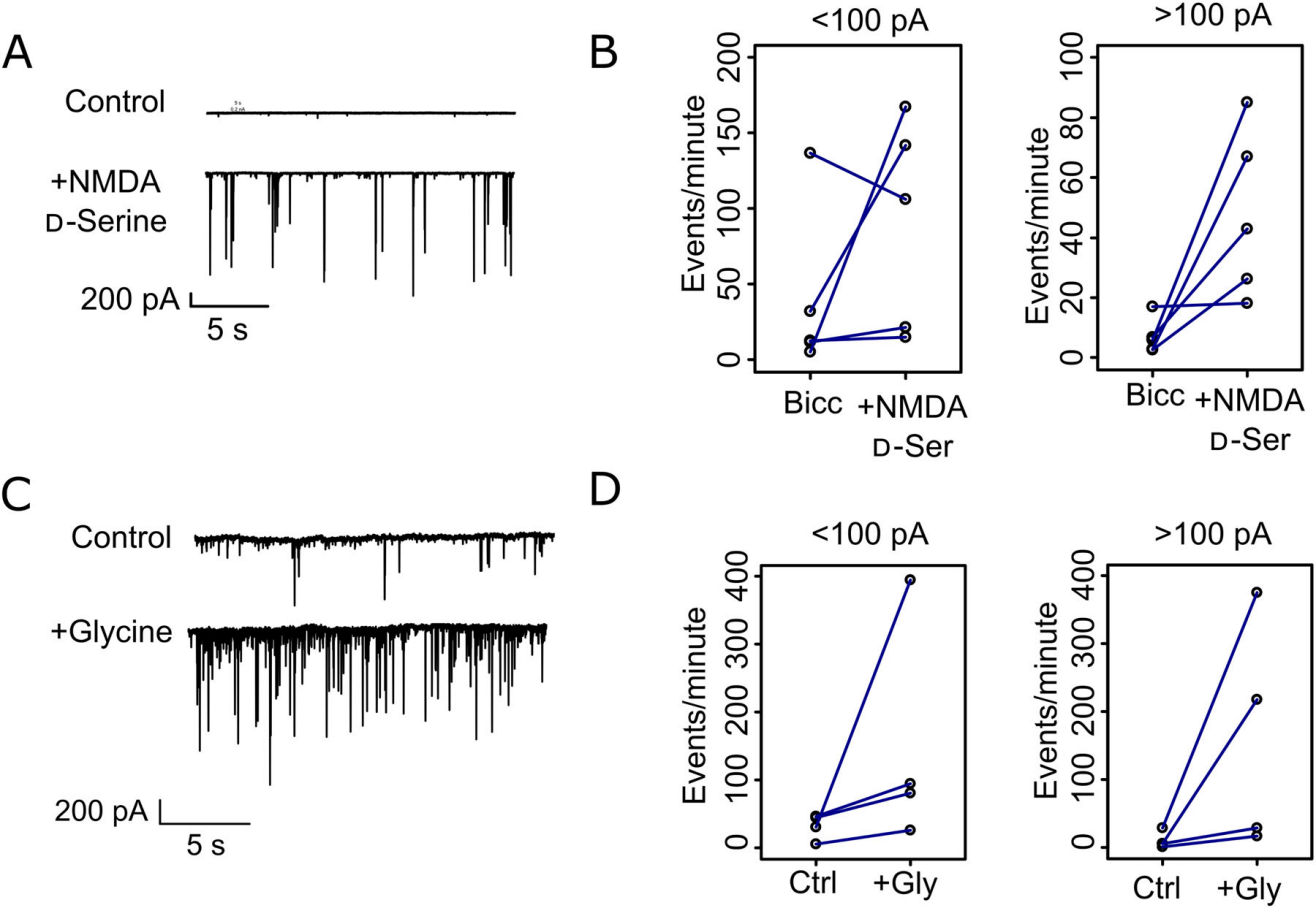
sEPSC recordings from vibrodissociated Purkinje cells showing NMDA or glycine alone (at 100 µM) increasing the frequency of both small (≤100 pA) and large (>100 pA) excitatory events *A*, recording from a vibrodissociated Purkinje cell showing segments of the record in the absence (upper trace) and presence (lower trace) of 50 µM NMDA and 10 µM d‐serine. The frequency of both small and large events can be seen to increase. *B*, the number of small (<100 pA) and large (>100 pA) amplitude events recorded per minute either before or after the application of NMDA for five neurons. In four out of five neurons the number of small events increased and for large events the number of events per minute increased in all five (five cells, five slices, three animals). *C*, recording from a vibrodissociated Purkinje cell showing segments of the record in the absence (upper trace) and presence (lower trace) of 100 µM glycine. The frequency of both small and large amplitude events can be seen to increase. *D*, the number of small and the number of large events per minute either before or after the application of glycine. Increases were seen for all four neurons from which recordings were made (four cells, four slices, three animals). All recordings were made in the presence of bicuculline (50 µM).

To examine this further, we assessed the effects of NMDA on small (≤100 pA) events and large (>100 pA) events. We found that whilst the biggest percentage change in event frequencies occurred for large events, in four of five neurons there was also an increase in the frequency of small events (Fig. 8).

### Slice recording confirms the glycine regulation of sEPSCs seen in vibrodissociated neurons

**Figure 9:**
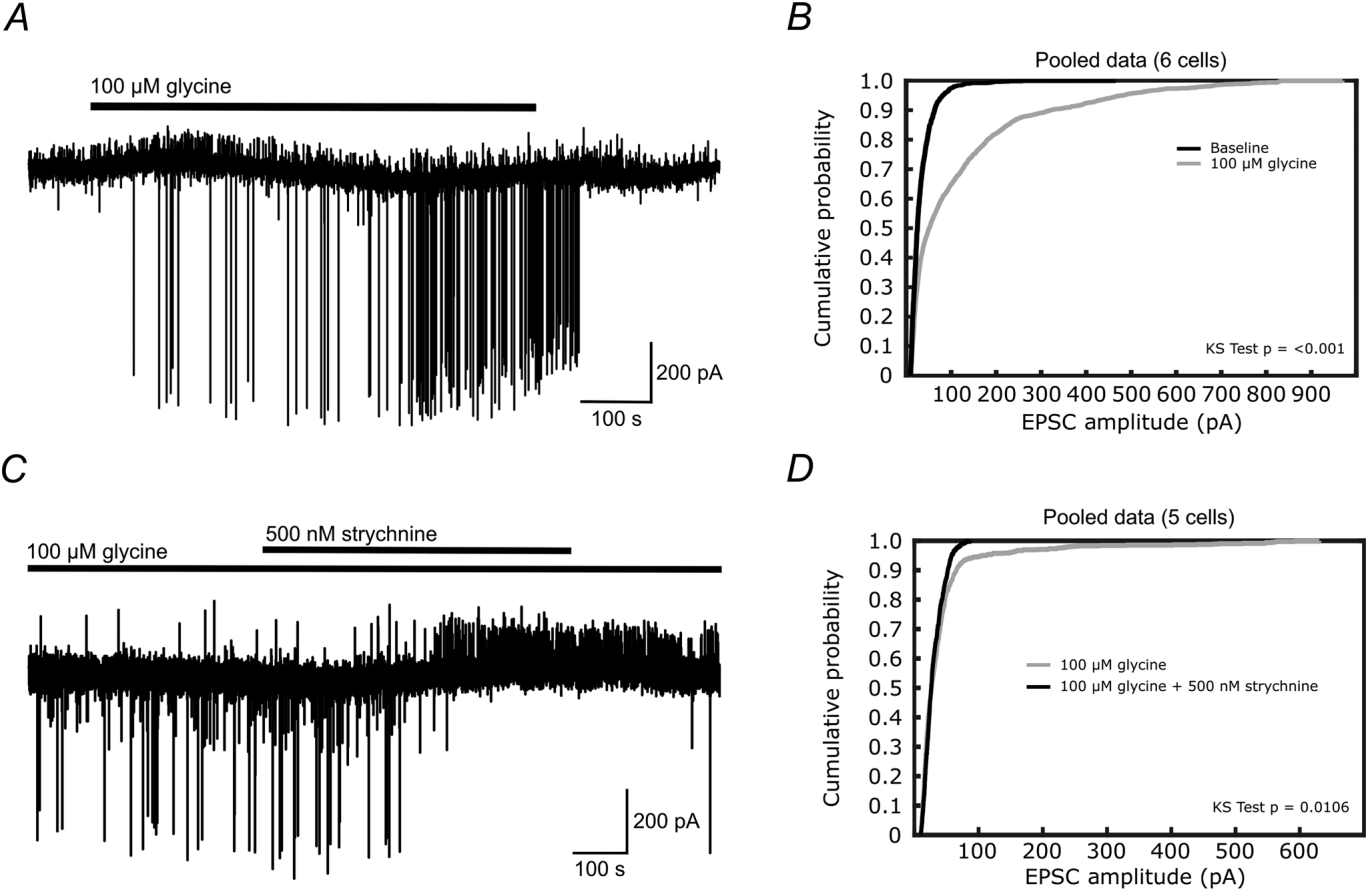
Cerebellar slice recordings reproduce the glycine receptor regulation seen in the nerve bouton preparation *A*, whole cell recording of Purkinje cell synaptic currents before, during and after the application of 100 µM glycine. *B*, cumulative distribution of amplitudes with and without glycine stimulation. A clear increase (*P* < 0.001, KS test) in large amplitude events is evident (six cell recordings from six separate slices and three animals). *C*, large sEPSCs are blocked in slices by the application of 500 nM strychnine, exactly as seen in the nerve bouton preparation. Strychnine also stimulated inhibitory (upward) currents in this neuron, but this latter effect was variable. *D*, cumulative amplitude histograms demonstrate the loss of large amplitude sEPSCs (*P* = 0.0106 KS test; *n* = 5 cells from five slices and four animals).

Finally, we examined whether this type of regulation might occur without the structural simplifications that are inherent to the nerve bouton preparation. To do this we used a cerebellar slice preparation with intact cytoarchitecture. Given the myriad of possible effects when applying NMDA to a cerebellar slice without TTX, we chose to apply 100 µM glycine instead, recording again in whole cell mode from the Purkinje neuron soma. In basal conditions, Purkinje neurons exhibited regular sEPSCs, which were almost exclusively <100 pA in amplitude (Fig. 9*A*, *B*). On application of 100 µM glycine, however, these neurons (11 of 12 tested) began to exhibit frequent, much larger sEPSCs, typically >100 pA, exactly as seen in the nerve bouton preparation. In a subset of recorded Purkinje neurons, we additionally added 500 nm strychnine hydrochloride, which led to a significant reduction in the large glycine‐induced sEPSCs (four of five neurons tested; Fig. 9*C*, *D*), whilst sparing the smaller sEPSCs observed in unstimulated conditions. Taken together these results suggest that pre‐synaptic glycine receptors can facilitate excitatory signalling in the intact cerebellum.

## Discussion

Our work points to previously unknown presynaptic receptor populations for both glycine and NMDA on the climbing fibre. The picture of NMDA receptor signalling has changed considerably in recent years and it is now known that the NMDA receptors can operate via a metabotropic pathway in addition to the ionotropic pathway with which they have been traditionally associated. This has expanded the discussion around the mechanisms by which NMDA can regulate presynaptic release (for an overview see e.g. Bouvier et al., 2018). However, the NMDA responses we see are blocked by Mg^2+^ and MK801. Both of these molecules act to physically block ion conduction via the pore and hence our observations are consistent with the idea that NMDA is acting via an ionotropic rather than a metabotropic pathway to produce the effects we see in this preparation. It has also been shown that assemblies of GluN1 and GluN3A NMDA receptor subunits can form glycine‐activated excitatory receptors (e.g. Chatterton et al., 2002; Grand et al., 2018). However, in our experiments this possibility is ruled out by the strychnine sensitivity, which is not seen in glycine‐operated NMDA receptors. Further, we deliberately used strychnine at a relatively low concentration, so that it will inhibit glycine receptor activity without major impacts on GABAergic transmission (Jonas et al., 1998). This strychnine sensitivity and the lack of Mg^2+^ block also rule out the possibility that glycine is somehow augmenting a residual glutamate/NMDA effect. Future work might extend these experiments to include the use of other agents, such as cyanotriphenylborate (CTB). Although CTB would need to be used with care, as it can block GABA_A_ receptors (Wooltorton et al., 1998), it is active against only certain subtypes of GlyRs, so it could potentially help in better defining the glycine receptor population.

Although it is not the main focus of our study, we found that NMDA increased the frequency of GABAergic sIPSCs produced by the isolated interneuron terminals that remain active and attached to vibrodissociated Purkinje cells. This finding is consistent with early work by Glitsch and Marty (1999) who found that the frequency of mIPSCs was increased following the application of NMDA. It also accords with later work by Duguid and Smart (2004) and Duguid et al. (2007) who found that presynaptic NMDA receptors mediated the effects of retrograde transmitter release from the Purkinje cell. Also consistent with a picture of presynaptic NMDA receptor regulation of interneuron transmitter release was the observation by Shin and Linden (2005) that NMDA caused a calcium rise in synaptic varicosities associated with stellate cell axons. Indeed, data from both Duguid and Smart (2004) and Glitsch (2008) show that at the rat interneuron–Purkinje cell synapses the Ca^2+^ influx through NMDA receptors alone is sufficient to account for increased GABA release. However, following on from this work a report by Christie and Jahr (2008) and another by Pugh and Jahr (2011) suggested a different picture.

As in earlier studies, Pugh and Jahr (2011) used a calcium imaging approach to examine presynaptic NMDA receptors in basket cell synapses and found no evidence for a calcium rise when l‐aspartate was applied to presynaptic terminals via electrophoresis. Furthermore, no calcium rise was seen in response to the local uncaging of glutamate. Finally, the same group found no evidence for presynaptic NMDA receptors at stellate cell synapses (Christie & Jahr, 2008). Nonetheless, more recent work by Rossi et al. (2012) did demonstrate presynaptic Ca^2+^ rises following the local uncaging of NMDA receptor agonists. A possible explanation for some of these varying results may stem from the age of the animals used. Christie and Jahr (2008) used animals at a later developmental stage (P15–P20), when there may well have been a down‐regulation of NMDA receptors during the later stages of development, as pointed out by Bouvier et al. (2015). However, Pugh and Jahr (2011) used P8–P18 animals, which covers quite a wide and important window, so further work may be necessary to understand these differences.

Our findings concerning presynaptic NMDA receptor regulation of sEPSCs are also part of a complicated picture. In the seminal paper of Glitsch and Marty (1999), no change in the frequency of mEPSCs was seen when NMDA was applied, implying that neither parallel fibre nor climbing fibre inputs were regulated by NMDA. However, in the nerve bouton preparation, applying NMDA with either 10 µM glycine or 10 µM d‐serine as a co‐agonist caused a substantial increase in the frequency of excitatory synaptic events (in 39 of 48 cells from 48 slices and 22 animals). One key difference between our work and earlier studies is that we studied all spontaneous, and not just mini, events. Na^+^ channels can be expressed at high levels in presynaptic boutons and indeed this has been shown to be the case for the parallel fibre–Purkinje cell synapse using electron microscopy (Caldwell et al., 2000). Therefore, it may be much easier to detect the effects of presynaptic receptors under our experimental conditions (i.e. in the absence of TTX; Fig. 7*B*).

Our data also show that high concentrations of glycine (100 µM) alone can modulate the frequency of excitatory transmitter release. This effect was substantially reduced by a low (500 nm) concentration of strychnine and was insensitive to the application of 1 mM Mg^2+^, suggesting that it is mediated by glycine, rather than NMDA, receptors. A similar effect of presynaptic glycine receptors has been described in other brain areas (Kubota et al., 2010; Lee et al., 2009; Ye et al., 2004). Further, in the cerebellum, Kawa (2003) demonstrated that glycine increased the frequency of spontaneous EPSCs recorded from Purkinje neurons in slices. However, Kawa (2003) observed that the glycine effect was TTX sensitive and thus suggested that the receptors involved were, probably, located on granule cells. We also found the effect of glycine to be sensitive to TTX, but a similar explanation can be discounted given the nature of the vibrodissociated cell preparation. Instead, our data suggest that, in this preparation, glycine receptors located either near release sites, or along the adhering axon, can depolarize the climbing fibre, activating voltage‐gated Na^+^ channels and hence generating action potential‐dependent release (Fig. 7*E*, *F*). In this context it is perhaps interesting to note that both NMDA and glycine can increase the frequency of events even in the presence of TTX (Fig. 7*C*, *F*) suggesting that, by depolarizing the membrane, they can activate voltage‐gated Ca^2+^ channels at release sites and/or increase Ca^2+^ release from internal stores (Duguid & Smart, 2004). It is also interesting to note that both NMDA and glycine receptors increase transmitter release, given their different ion selectivity. A possible reason for this is that chloride concentrations in nerve terminals and/or the climbing fibre fragments are higher than in the soma, and thus glycine receptors can have a depolarizing effect (e.g. Khirug et al., 2008; Kunz et al., 2012; Price & Trussell, 2006).

Looking at the distribution of amplitudes of synaptic currents stimulated either by NMDA or by high concentrations of glycine alone can provide insight concerning the type(s) of excitatory synapse being regulated. In particular, in many cases the largest events were greater than 500 pA (43 out of 46 responding cells from, 46 slices and 27 animals). This is more than 10 times the size of currents expected from a typical parallel fibre–Purkinje cell synapse so these events seem very unlikely to arise from such connections as this would require coordinated synaptic release. On the other hand, large amplitude synaptic currents driven by action potentials are the hallmark of climbing fibre connections. In keeping with this idea, we have shown that vibrodissociated neurons stain positively for the climbing fibre marker peripherin and thus appear to retain climbing fibre inputs. We have also shown that the very largest events are blocked by the application of TTX. The variable size of climbing fibre fragments that remain attached to the vibrodissociated cells may generate some of the cell‐to‐cell variability seen in these largest event amplitudes. Interestingly, although the largest events in any cell recording were always eliminated by the application of TTX, it was also evident that for some cells, a few events, even in the presence of TTX, occasionally reached ∼400 pA in size and are thus also much larger than even the largest parallel fibre synaptic currents seen by Isope and Barbour (2002). We suggest that the depolarizing action of NMDA and/or glycine receptors is likely to explain this. Thus, in the presence of TTX, the depolarization induced by activating these ligand‐gated ion channels would, in turn, activate voltage‐sensitive calcium channels, creating local calcium rises in the climbing fibre that could trigger large events.

Our data raise additional possibilities in relation to the presynaptic regulation of parallel fibre release. The numbers of both large (>100 pA) and small (<100 pA) excitatory synaptic currents increase when using either glycine or NMDA receptors to raise release frequency (Fig. 8). One explanation for the increase in small amplitude events is that these receptors may also be able to regulate the release of transmitter at parallel fibre terminals. For NMDA this idea is consistent with other literature reports (e.g. Lonchamp et al., 2012). However, in the vibrodissociated preparation we do not expect to retain large numbers of parallel fibre terminals and it must be remembered that if vesicles were released individually by the climbing fibre, then they could generate a synaptic current of very similar size (Silver et al., 1998).

The source of glycine for receptor regulation is currently unknown. In a similar situation at the calyceal synapse in the medial nucleus of the trapezoid body (MNTB), presynaptic glycine receptors have been shown to regulate the release of glutamate. However, in that case MNTB neurons receive direct glycinergic input and high levels of activity in these afferents lead to activation of the presynaptic glycine receptors via transmitter spillover (Turecek & Trussell, 2001). Cerebellar Purkinje cells do not receive glycinergic input so a similar explanation is ruled out. However, it has been shown that Bergmann glia, which encapsulate the Purkinje cell, express the GlyT1 transporter and that ‘reverse’ transport of glycine by GlyT1 might regulate glycine concentrations in the vicinity of the neuron (Huang et al., 2004). This has therefore been suggested as the source of glycine in NMDA modulation of the interneuron–Purkinje cell IPSCs. It is also possible that this mechanism might promote the modulation of presynaptic glycine receptors. Whatever the exact mechanism, a subthreshold modulatory effect of glycine receptor activation could occur at concentrations well below the 100 µM level used here to trigger action potentials. Indeed, we sometimes saw a small effect of glycine at 10 µM, which first prompted us to test d‐serine as a co‐agonist. The EC_50_ values for glycine receptor activation are reported to be in the range of ∼20–150 µM and are dependent on subunit composition and splicing (Miller et al., 2004; Raltschev et al., 2016; Sanchez et al., 2015). Further, since glycine receptors are not blocked by Mg^2+^ they are not restricted to activity‐dependent modulation, which the presynaptic NMDA receptors on the climbing fibre will be. Indeed, if the glycine receptors have an excitatory role, then they could even help in partially alleviating NMDA receptor block.

In summary, our work provides the first evidence that both presynaptic glycine and NMDA receptors can regulate transmission at the climbing fibre–Purkinje cell synapse in young animals. Interestingly, given that the influence of these receptors is much more pronounced in the absence of TTX, similar regulation may be underestimated in other parts of the CNS if examined only via changes in mini event frequency. At present the physiological role of these receptors is uncertain. Clearly the NMDA receptors may have a role as autoreceptors but it will also be of interest to know whether they are activated by retrograde transmitter release. In addition, given the age of animal used in our studies (P10), it is tempting to speculate that both NMDA and glycine receptors may be important in the complex maturation of the climbing fibre innervation profile on Purkinje cells (Hashimoto & Kano, 2013). Further work will be needed to address these issues.

## Additional information

## Competing interests

The authors declare no competing interests.

## Author contributions

D.C.H.B., A.D.R., M.C., L.I., Y.P. and G.W.J.M. acquired the data. D.C.H.B., A.D.R., M.C., G.M. and G.W.J.M. performed the analysis. G.W.J.M., D.C.H.B., A.D.R and T.G.S. designed the experiments; G.W.J.M. wrote the first draft of the paper and all authors provided critical comments relevant to intellectual content.

## Funding

This work was part‐funded by BBSRC grant BB/D01817X/1 (G.W.J.M. and T.G.S.) and by a studentship from the Medical Research Council (A.D.R.) and an Emerging Leader Fellowship from Epilepsy Research Institute UK (F2102 Morris; G.M.) and by the Royal Society (RGS∖R2∖222326; G.M.).

## Author's present address

Yuriy Pankratov: Department of Biological Sciences, University of Warwick, Coventry, UK.

## Supporting information


Peer Review History

## Data Availability

The data reported in this study are available on request from the corresponding author, G.W.J.M. or, for slice work, G.M.
