# Supplementary material for: NMDA and glycine receptors provide dual presynaptic regulation on climbing fibre inputs to cerebellar Purkinje cells
Source: J Physiol. 2026 May 24;604(12):4926–44. doi: 10.1113/JP287456 (PMC13267686; doi:10.1113/JP287456)
Supplement: Supplementary file 1 — Peer Review History [file TJP-604-4926-s001.pdf]

The Journal of Physiology

NMDA and Glycine receptors provide dual presynaptic regulation on climbing fibre inputs to cerebellar Purkinje cells.

Guy WJ Moss, David CH Benton, Alan D. Robertson, Matthew Caldwell, Yuiry Pankratov, Lema Imam, Trevor G. Smart, and Gareth Morris
DOI: 10.1113/JP287456

Corresponding author(s): Guy Moss (g.moss@ucl.ac.uk)

The following individual(s) involved in review of this submission have agreed to reveal their identity: Stefan F Boehm (Referee #2)

Review Timeline:

Submission Date:	11-Oct-2024
Editorial Decision:	21-Nov-2024
Revision Received:	05-Feb-2026
Editorial Decision:	18-Mar-2026
Revision Received:	13-Apr-2026
Accepted:	17-Apr-2026

Senior Editor: Katalin Toth

Reviewing Editor: Samuel Young

Transaction Report:

(Note: With the exception of the correction of typographical or spelling errors that could be a source of ambiguity, letters and reports are not edited. Depending on transfer agreements, referee reports obtained elsewhere may or may not be included in this compilation. Referee reports are anonymous unless the Referee chooses to sign their reports.)

Dear Dr Moss,

Re: JP-RP-2024-287456 "NMDA and Glycine receptors provide dual presynaptic regulation on climbing fiber inputs to cerebellar Purkinje cells." by Guy WJ Moss, David CH Benton, Alan D. Robertson, Matthew Caldwell, Yuiry Pankratov, Lema Imam, Trevor G. Smart, and Gareth Morris

Thank you for submitting your manuscript to The Journal of Physiology. It has been assessed by a Reviewing Editor and by 2 expert referees and we are pleased to tell you that it is potentially acceptable for publication following satisfactory major revision.

Please advise your co-authors of this decision as soon as possible.

The referee reports are copied at the end of this email.

Please address all the points raised and incorporate all requested revisions or explain in your Response to Referees why a change has not been made. We hope you will find the comments helpful and that you will be able to return your revised manuscript within 9 months. If you require longer than this, please contact journal staff: jp@physoc.org. Please note that this letter does not constitute a guarantee for acceptance of your revised manuscript.

Your revised manuscript should be submitted online using the link in your Author Tasks: Link Not Available. This link is accessible via your account as Corresponding Author; it is not available to your co-authors. If this presents a problem, please contact journal staff (jp@physoc.org). Image files from the previous version are retained on the system. Please ensure you replace or remove any files that are being revised.

If you do not wish to submit a revised version of your manuscript, you must inform our journal staff (jp@physoc.org) or reply to this email to request withdrawal. Please note that a manuscript must be formally withdrawn from the peer review process at one journal before it may be submitted to another journal.

TRANSPARENT PEER REVIEW POLICY: To improve the transparency of its peer review process, The Journal of Physiology publishes online as supporting information the peer review history of all articles accepted for publication. Readers will have access to decision letters, including Editors' comments and referee reports, for each version of the manuscript, as well as any author responses to peer review comments. Referees can decide whether or not they wish to be named on the peer review history document.

ABSTRACT FIGURES: Authors are expected to use The Journal's premium BioRender account to create/redraw their Abstract Figures. Information on how to access this account is here: https://physoc.onlinelibrary.wiley.com/journal/14697793/biorender-access.

This will enable Authors to create and download high-resolution figures. If authors have used the free BioRender service, they can use the instructions provided in the link above to download a high-resolution version suitable for publication.

The link provided should only be used for the purposes of this submission. Authors will be charged for figures created on this account if they are not related to this manuscript submission.

LANGUAGE EDITING AND SUPPORT FOR PUBLICATION: If you would like help with English language editing, or other article preparation support, Wiley Editing Services offers expert help, including English Language Editing, as well as translation, manuscript formatting, and figure formatting at www.wileyauthors.com/eeo/preparation. You can also find resources for Preparing Your Article for general guidance about writing and preparing your manuscript at www.wileyauthors.com/eeo/prepresources.

RIGOUR AND REPRODUCIBILITY: We recommend authors consult The Journal's Rigour and Reproducibility requirements to ensure that their manuscript is suitable for publication: https://physoc.onlinelibrary.wiley.com/pb-assets/hub-assets/physoc/documents/TJP-Rigour-and-Reproducibility-Requirements.pdf

REVISION CHECKLIST:

Check that your Methods section conforms to journal policy: https://jp.msubmit.net/cgi-bin/main.plex?form_type=display_requirements#methods.

Check that data presented conforms to the statistics policy: https://jp.msubmit.net/cgi-bin/main.plex?form_type=display_requirements#statistics.

Upload a full Response to Referees file. To create your 'Response to Referees': copy all the reports, including any comments from the Senior and Reviewing Editors, into a Microsoft Word, or similar, file and respond to each point, using font or background colour to distinguish comments and responses and upload as the required file type.

Please upload two versions of your manuscript text: one with all relevant changes highlighted and one clean version with no changes tracked. The manuscript file should include all tables and figure legends, but each figure/graph should be uploaded as separate, high-resolution files.

You may also upload:

- 'Potential Cover Art' for consideration as the issue's cover image
- Appropriate Supporting Information (Video, audio or data set: see https://jp.msubmit.net/cgi-bin/main.plex?form_type=display_requirements#supp).

We look forward to receiving your revised submission.

If you have any queries, please reply to this email and we will be pleased to advise.

Yours sincerely,

Katalin Toth
Senior Editor
The Journal of Physiology

REQUIRED ITEMS:

- The contact information for the person responsible for 'Research Governance' at your institution needs to be provided. This includes their name and an institutional email address. Please ensure the contact is not an author on this paper and provide an alternate contact if necessary, or confirm in the submission form that the author whose email was provided has sole responsibility for research governance. This is the person who is responsible for regulations, principles and standards of good practice in research carried out at the institution, for instance the ethical treatment of animals, the keeping of proper experimental records or the reporting of results.

EDITOR COMMENTS

Reviewing Editor:

Comments to the Author:
This manuscript focuses on the characterization of the functional role of presynaptic NMDA and glycine receptors in climbing fiber inputs in acutely isolated Purkinje Cell neurons. Both reviewers found that the data was rigorous, made novel observations, conclusions drawn were largely supported by the data. Both reviewers felt the findings would be highly impactful. However, both reviewers had concerns, and the authors will need to address these with experiments, reanalysis of data, and rewriting of the text. In particular, the authors should carry out experiments that use alternative GlyR agonists or antagonists to strengthen their conclusions of presynaptic GlyRs. The authors need to further carry out analysis of amplitude to go with time courses that are analyzed Figures 3,4,5. In addition, the authors should show in cell comparisons in Figu 3C and 3F as demonstrated in Figure 8. The authors need to carefully revise and rewrite their manuscript and provide more in-depth discussion of their results based on the positive comments by the reviewers. Finally, there are missing details about animals with respect to ethical approval, on access to food and water and how animals are euthanized, this is required per Journal policy.

REFEREE COMMENTS

Referee #1:

This manuscript describes the presence and function of NMDA and glycine receptors on synaptic inputs to acutely isolated Purkinje neurons. The work provides compelling evidence that these are present and points to ways they might more readily be found on other terminals. It does leave some questions around how these receptors actually function and may give some erroneous indications of their actual functions. I outline criticisms below.

The findings that NMDA and glycine receptors are present on presynaptic terminals is not new, but that they are present on climbing fiber presynapses is. The finding does have reasonably strong implication for understanding integration of cerebellar signaling. The conclusions are reasonable but a more complete discussion of the meaning of the findings is needed.

1) The data that both NMDA receptor activation and glycine receptor activation can lead to action potentials is reasonably argued. But it is important to note that it is not clear how they function in intact cerebellum. This is especially true in the isolated Purkinje cells where both climbing fibers and local inhibition are mere fragments, but also in slices where axons of climbing fibers are cut. These may change the ratio of Na and K conductances in the remaining axons or bias Cl- pumping effectivity, or even change the membrane potential to make it more likely that an inward Cl- current can achieve threshold. For NMDA receptors it is telling that Mg largely blocks the large amplitude events indicating that glutamate is unlikely to trigger action potentials in physiological conditions. In addition, it seems unlikely that Bergmann glia glycine pumps would generate 100 µM glycine or even close to that. It would seem much more likely that both NMDA receptors and glycine receptors will modulate activity dependent synaptic responses in vivo. In this case it is likely that glycine receptors mediate different effects than NMDA. If, for example, lower levels of glycine open sub-threshold Cl- conductances, they are likely to modify the action potential shape and perhaps its dwell time around peak Ca2+ channel current flow. I am uncertain of what the effect will be on synaptic release, but it does bear discussion. NMDA receptor effects may depend on either direct action potential depolarization and loss of Mg2+ block or on Ca2+ entry mediating changes in presynaptic Ca2+. In either case it seems unlikely to me that the physiological effect of agonists will be to drive action potentials. I am not sure that is the conclusion the authors feel is true either, but a more detailed discussion of the implications of these receptors would provide some clarity.

The effect of Mg against NMDA events is telling, because it seems clear that in physiological conditions NMDA cannot generate a large event.

2) If NMDA or glycine are triggering action potentials - which I believe the authors have shown - then Ca2+ transients in the FM labeled boutons should be recordable. It would have been very convincing to see such transients. They could be selectively labeled with a low affinity am-ester Ca2+ dye with a high postsynaptic EGTA or BAPTA concentration to effectively prevent postsynaptic signals.

3) The choice of DNQX to block AMPA receptors selectively is unfortunate. This is an NMDA receptor glycine site inhibitor as well as an AMPA/kainate antagonist. I think the block will be overcome by raised (10 µM) glycine concentrations, but it generates some confusion in the results.

4) It would have been useful to see the kinetics of the events. That is to say an expansion of the time base to show example events. That way the clear large events could be interpreted and the different time courses of AMPA and GABA mediated postsynaptic events separated. The one figure showing separation of these currents is not at all convincing. Only two inward events are visible and the choice of a +5mV holding potential seems strange.

5) The effect of 4-AP in driving events and its lack of block by Mg seem very hard to interpret. If 4-AP is driving a substantial increase in activity I would think that it causes a depolarization in presynaptic resting potential as well as a lengthening of the presynaptic spike. All this would mean that NMDA receptor block by Mg would be lost anyway during the activity, and I would not expect it to make much of a difference at all under any circumstances. This does not mean that the effect of NMDA is not presynaptic it just means that 4-AP might not be useful.

6) The effect of NMDA and glycine acting through climbing fibers is considered to be why event sizes are large. In all cases I would therefore expect NMDA and glycine to cause large events. However, only the time courses are analyzed in figures 3,4 and 5. Why not show the amplitude data as well.

7) Metabotropic glycine receptors have been identified (a previously orphan receptor GPR158). It seems they are worth discussing in this context

Minor points

The statistics to uncover what appear to be two populations of synaptic inputs seem unnecessary given that they can be pharmacologically separated.

For the exponential fits in figure 2B a clearer way of showing the two exponentials would have been helpful. Maybe different colors instead of dashed lines.

On line 728 the authors note that NMDA receptors might be activated by retrograde transmitter release. Would it not be more likely that they are autoreceptors at glutamate release sites?

Referee #2:

The manuscript by David Benton et al. entitled "NMDA and Glycine receptors provide dual presynaptic regulation on climbing fiber inputs to cerebellar Purkinje cells" describes presynaptic NMDA and glycine receptors activation of which raised frequencies and amplitudes of spontaneous EPSCs (and IPSCs) in vibrodissected cerebellar Purkinje neurons. NMDAR- and GlyR-induced EPSCs were identified as originating from climbing fibers by their amplitudes and by staining of adhering neurites for peripherin. A large fraction of these induced EPSCs were blocked by TTX. Effects of glycine were confirmed in cerebellar slices. From these results, the authors conclude that "NMDA and glycine receptors can both operate as presynaptic regulators of climbing fiber excitatory transmitter release". These results might be interesting to the audience of The Journal of Physiology, but some of the conclusions are not fully supported by the data as outlined below.

Major issues:

A) Effects are claimed to be mediated by presynaptic receptors: the results obtained with TTX argue against this conclusion. A presynaptic receptor is located at presynaptic terminals; accordingly, a depolarization caused by the opening of such receptors can be expected to depolarize the terminal which will lead to calcium influx and exocytosis even without any propagation of action potentials. For this reason, presynaptic ionotropic receptors increase frequencies of miniature EPSCs and IPSCs which are determined with Na+ channels being blocked. Therefore, when transmitter release caused by the activation of a receptor is blocked by TTX, this receptor is unlikely to be located at the presynaptic terminal itself but rather somewhere at the adhering axon. In this context, see lines 671 to 679; this needs to be rephrased.

B) The authors describe the distribution of interevent intervals by a biexponential fit. It remains entirely unclear whether this distribution has any functional relevance, and the authors refrain from explaining this finding. Accordingly, this fitting could be omitted. One would rather wish to see direct experimental results (instead of fitted data), such as cumulative probability plots (as in figure 9) for both, interevent intervals and amplitudes. Likewise, instead of normalized data (e.g. figure 3C and F) it would be preferable to compare results as obtained by using spaghetti plots showing in-cell comparisons (as in figure 8b and d).

C) The partial block of glycine evoked responses by 0.5 µM strychnine argues in favor of presynaptic GlyRs. Nevertheless, this conclusion needs to be strengthened to be unequivocal. Accordingly, either alternative agonists (e.g. beta-alanine or taurine) and/or antagonists (e.g. cyanotriphenylborate) should be tested in addition.

Minor points:

Line 103: "...IPSC effects were largely independent of TTX". Do you mean insensitive towards TTX?

Line 284 (legend to figure 1): "E)" instead of "D)".

Lines 612 and 613: "The picture of NMDA receptor signalling has changed considerably over in recent years...". Omit "over".

Lines 734 to 744: The last paragraph is too speculative and can be omitted.

END OF COMMENTS

Reply.

Sincere apologies for the delay in replying which was due to difficult personal circumstances. We thank the reviewers and editor for many thoughtful comments. In response to their suggestions we have made a number of changes to the figures and text that have helped to strengthen our manuscript. Our replies, along with the specific points raised by the reviewers, are given below.

REFEREE COMMENTS

Referee #1:

This manuscript describes the presence and function of NMDA and glycine receptors on synaptic inputs to acutely isolated Purkinje neurons. The work provides compelling evidence that these are present and points to ways they might more readily be found on other terminals. It does leave some questions around how these receptors actually function and may give some erroneous indications of their actual functions. I outline criticisms below.

The findings that NMDA and glycine receptors are present on presynaptic terminals is not new, but that they are present on climbing fiber presynapses is. The finding does have reasonably strong implication for understanding integration of cerebellar signaling. The conclusions are reasonable but a more complete discussion of the meaning of the findings is needed.

1) The data that both NMDA receptor activation and glycine receptor activation can lead to action potentials is reasonably argued. But it is important to note that it is not clear how they function in intact cerebellum.

This is especially true in the isolated Purkinje cells where both climbing fibers and local inhibition are mere fragments, but also in slices where axons of climbing fibers are cut.

These may change the ratio of Na+ and K+ conductances in the remaining axons or bias Cl- pumping effectivity, or even change the membrane potential to make it more likely that an inward Cl- current can achieve threshold.

For NMDA receptors it is telling that Mg largely blocks the large amplitude events indicating that **glutamate is unlikely to trigger action potentials in physiological conditions.** In addition, it seems unlikely that Bergmann glia glycine pumps would generate 100 µM glycine or even close to that. It would seem much more likely that both **NMDA receptors and glycine receptors will modulate activity dependent synaptic responses in vivo.** In this case it is likely that glycine receptors mediate different effects than NMDA. If, for example, lower levels of glycine open sub-threshold Cl- conductances, they are likely to modify the action potential shape and perhaps its dwell time around peak Ca2+ channel current flow. I am uncertain of what the effect will be on synaptic release, but it does bear discussion.

NMDA receptor effects may depend on either direct action potential depolarization and loss of Mg2+ block or on Ca2+ entry mediating changes in presynaptic Ca2+. In either case it seems unlikely to me that the physiological effect of agonists will be to drive action potentials. **I am not sure that is the conclusion the authors feel is true either,** but a more detailed discussion of the implications of these receptors would provide some clarity.

The effect of Mg against NMDA events is telling, because it seems clear that in physiological conditions NMDA cannot generate a large event.

We appreciate these thoughtful comments and agree that the role of these receptors in vivo is unclear. We used a reductionist preparation to help identify the location of the GlyRs and NMDARs, rather than to determine their in vivo function, which would need to be the basis of another study. Indeed, we tried to avoid making specific claims about how these receptors function in the intact cerebellum and said "At present the physiological role of these receptors is unclear". However, we agree that it would be helpful to discuss the possibilities raised by the reviewer and we have now included these ideas in our revised manuscript. We also now explicitly point out that a sub threshold activation of glycine receptors at concentrations substantially below 100 uM could exert a modulatory effect on the climbing fiber. Further, if the glycine receptors are acting in an excitatory fashion in vivo, then they could also assist in relieving Mg^{2+} block of the NMDA receptors.

2) If NMDA or glycine are triggering action potentials - which I believe the authors have shown - then Ca2+ transients in the FM labeled boutons should be recordable. It would have been very convincing to see such transients. They could be selectively labeled with a low affinity am-ester Ca2+ dye with a high postsynaptic EGTA or BAPTA concentration to effectively prevent postsynaptic signals.

We appreciate the point made by this reviewer, and indeed we considered such experiments during the work described here. However, Ca^{2+} measurements would only provide a proxy for excitation in the terminals and we have already demonstrated the presence of action potentials which is a more direct measure.

3) The choice of DNQX to block AMPA receptors selectively is unfortunate. This is an NMDA receptor glycine site inhibitor as well as an AMPA/kainate antagonist. I think the block will be overcome by raised (10 µM) glycine concentrations, but it generates some confusion in the results.

It is true that not only DNQX, but also CNQX (Lester et al., 1989), can compete for the NMDA glycine binding site. However, these remain very popular antagonists because, as the reviewer points out, this effect would be significantly reduced by competition with raised glycine (or serine) concentrations. If DNQX were preventing NMDA receptor activation, we would not be able to get NMDA responses as we do with both glycine and D-serine as co-agonists, along with block by Mg^{2+} and/or MK801. For these reasons we don't think this substantially affects the interpretation of our results.

4) It would have been useful to see the kinetics of the events. That is to say an expansion of the time base to show example events. That way the clear large events could be interpreted and the different time courses of AMPA and GABA mediated postsynaptic events separated.

The one figure showing separation of these currents is not at all convincing. Only two inward events are visible and the choice of a +5mV holding potential seems strange.

We have replaced the trace showing different time courses, which hopefully is now much clearer. Because we have near symmetry in anions and cations, events only identifiably “split” close to 0 mV, hence the choice of holding potential (which is now zero in the revised manuscript).

5) The effect of 4-AP in driving events and its lack of block by Mg seem very hard to interpret. If 4-AP is driving a substantial increase in activity I would think that it causes a depolarization in presynaptic resting potential as well as a lengthening of the presynaptic spike. All this would mean that NMDA receptor block by Mg would be lost anyway during the activity, and I would not expect it to make much of a difference at all under any circumstances. This does not mean that the effect of NMDA is not presynaptic it just means that 4-AP might not be useful.

There seems to be some misunderstanding here and obviously we were not sufficiently clear. We fully expect 4-AP to depolarise the terminals. The 4-AP experiment was designed to show that the Mg^{2+} effects that occur when NMDA is applied are effects due to block of the NMDA receptor rather than unwanted impacts on some other aspect of synaptic transmission. To test this idea we took NMDA ‘out of the equation’ and triggered transmitter release with 4-AP alone, then added Mg^{2+}, which, predictably, had virtually no effect. It was never our intension to have any effect on NMDA receptors and no glutamate or NMDA was applied while the cells were perfused with 4-AP. We apologise for the confusion and have tried to make this clearer in the revised text.

6) The effect of NMDA and glycine acting through climbing fibers is considered to be why event sizes are large. In all cases I would therefore expect NMDA and glycine to cause large events. However, only the time courses are analyzed in figures 3,4 and 5. Why not show the amplitude data as well.

We were not entirely clear what is being requested by the reviewer here as there are two potential issues. First, there is the frequency of large amplitude events, which increases dramatically following the application of either NMDA or glycine. Hopefully this is clear from the raw current traces that we have shown in our figures.   However, a second point relates to the relative frequency of such events. Depending on the resting potential and conductances of the climbing fiber fragments the expected increase in the proportion of large events is much less certain. Be that as it may, prompted by the reviewer, we have now added cumulative amplitude plots to a number of our figures as an aid to further understanding the amplitude data.

7) Metabotropic glycine receptors have been identified (a previously orphan receptor GPR158). It seems they are worth discussing in this context

This is an interesting topic, although, as the reviewer doubtless knows, metabotropic glycine receptors are not strychnine sensitive (Laboute et al., Science 2023). Nonetheless, for completeness we now mention this point in the revised manuscript along with a comment about the reports on excitatory glycine (NMDA) receptors.

Minor points

The statistics to uncover what appear to be two populations of synaptic inputs seem unnecessary given that they can be pharmacologically separated.

We were probably not clear enough here. What our statistical analysis uncovers is the presence of bursting behaviour amongst the synapses contacting the vibrodissociated Purkinje neurons. The two components present will not automatically map to excitatory and inhibitory synaptic inputs. However, because we use changes in event frequency as a key marker of presynaptic effects the observation of bursting behaviour is important to address when calculating inter-event time constants. This is what we attempted to illustrate.

For the exponential fits in figure 2B a clearer way of showing the two exponentials would have been helpful. Maybe different colors instead of dashed lines.

As suggested by the reviewer we have now included coloured lines to show the individual exponential components more clearly.

On line 728 the authors note that NMDA receptors might be activated by retrograde transmitter release. Would it not be more likely that they are autoreceptors at glutamate release sites?

We accept the point that it is possible for these receptors to act as autoreceptors and we have now added this point to the discussion. However, we have previously shown (Duguid and Smart, 2004) that presynaptic NMDARs are activated by retrograde glutamate in the cerebellum, so we include this point too.

Referee #2:

The manuscript by David Benton et al. entitled "NMDA and Glycine receptors provide dual presynaptic regulation on climbing fiber inputs to cerebellar Purkinje cells" describes presynaptic NMDA and glycine receptors activation of which raised frequencies and amplitudes of spontaneous EPSCs (and IPSCs) in vibrodissected cerebellar Purkinje neurons. NMDAR- and GlyR-induced EPSCs were identified as originating from climbing fibers by their amplitudes and by staining of adhering neurites for peripherin. A large fraction of these induced EPSCs were blocked by TTX. Effects of glycine were confirmed in cerebellar slices. From these results, the authors conclude that "NMDA and glycine receptors can both operate as presynaptic regulators of climbing fiber excitatory transmitter release". These results might be interesting to the audience of The Journal of Physiology, but some of the conclusions are not fully supported by the data as outlined below.

Major issues:

A) Effects are claimed to be mediated by presynaptic receptors: the results obtained with

TTX argue against this conclusion. A presynaptic receptor is located at presynaptic terminals; accordingly, a depolarization caused by the opening of such receptors can be expected to depolarize the terminal which will lead to calcium influx and exocytosis even without any propagation of action potentials. For this reason, presynaptic ionotropic receptors increase frequencies of miniature EPSCs and IPSCs which are determined with Na+ channels being blocked. Therefore, when transmitter release caused by the activation of a receptor is blocked by TTX, this receptor is unlikely to be located at the presynaptic terminal itself but rather somewhere at the adhering axon. In this context, see lines 671 to 679; this needs to be rephrased.

First, we should perhaps point out that the application of either glycine or NMDA in the presence of TTX can still increase event frequency above baseline (Fig. 7). Although this effect is much smaller than that in the absence of TTX, this response is exactly what the reviewer suggests should be the case for a presynaptic location. It is also an observation that is consistent with our immunohistochemistry for the NMDA receptor. Whether or not these presynaptic receptors could activate Na^+ channels in vivo, is certainly open to question and we have tried not to suggest this happens. However, we believe it should not be entirely ruled out as it has been known for many years that synaptosomes contain sodium channels (e.g. Jover et al., 1988. J. Biol. Chem. Jan 25;263(3):1542-8). In addition, we cite a paper by Caldwell et al., (2000) which shows that Nav1.6 channels are packed at a high density within presynaptic terminals contacting Purkinje cells. Also, because climbing fibers make multiple contact points with their Purkinje cell partners, this could help in driving towards an action potential threshold. Having said all that, we certainly accept the reviewer's point that the location could be axonal for glycine receptors. Therefore, as suggested by the reviewer, we have rephrased our paper to specifically point out that an axonal location is entirely possible and to discuss the point about activation of voltage-gated Ca^{2+} channels (lines 526-532).

B) The authors describe the distribution of interevent intervals by a biexponential fit. It remains entirely unclear whether this distribution has any functional relevance, and the authors refrain from explaining this finding. Accordingly, this fitting could be omitted. One would rather wish to see direct experimental results (instead of fitted data), such as cumulative probability plots (as in figure 9) for both, interevent intervals and amplitudes. Likewise, instead of normalized data (e.g. figure 3C and F) it would be preferable to compare results as obtained by using spaghetti plots showing in-cell comparisons (as in figure 8b and d).

We no longer show normalised data and now provide spaghetti plots for Figures 3C and 3F (3C and 3G in our revised manuscript) as requested by the referee. We have also now added a number of cumulative plots that we hope are helpful and an additional spaghetti plot in Fig. 7F.

<u>The biexponential distribution of intervals</u>. We use changes in event frequency as the hallmark of presynaptic effects throughout our study. For this reason, we feel it important to show how these frequencies were calculated and hence to point out that the time between events is sometimes biexponentially distributed. This allows readers to understand why it is appropriate to use a weighted time constant as a measure of event frequency. We have tried to make this clearer in our revised manuscript. If one can accept that the correct calculation of the inter-event interval is important to illustrate, then there is also the matter of how to display such a distribution to show that it is biexponential. We selected what we believe is the gold standard for the display of intervals. It was developed by Sigworth and Sine (paper cited in our manuscript) as an improved method of displaying intervals taken from the field of single channel recording where the mathematically equivalent problem

presents itself. Not only is this display method much clearer than other methods when exponential time constants are not well-separated, but it is also more statistically sound in showing the goodness of fit because of its use of a square root y-axis. These advantages over some of the other approaches are detailed in the Sigworth and Sine paper. For these reasons we would like to make the case for keeping the distribution data plotted as we have done, but we have tried to make our reasoning clearer in the revised manuscript.

C) The partial block of glycine evoked responses by 0.5 µM strychnine argues in favor of presynaptic GlyRs. Nevertheless, this conclusion needs to be strengthened to be unequivocal. Accordingly, either alternative agonists (e.g. beta-alanine or taurine) and/or antagonists (e.g. cyanotriphenylborate) should be tested in addition.

We appreciate this thought. However, we have shown that cyanotriphenylborate (CTB) blocks $GABA_A$ receptors (see Cyanotriphenylborate is an uncompetitive inhibitor of human $GABA_A$ recombinant receptors, March 1998, British Journal of Pharmacology 123:U30-U30). It is also known that CTB blocks only some subtypes of GlyRs. For these reasons we feel CTB results would not add great clarity. Taurine is also not particularly selective as, for example, it directly interacts with the NMDA receptor (e.g. Suárez, L.M. and Solís, J.M., 2006; Chan et al., 2013).

We have demonstrated glycine agonism with strychnine block (at low concentrations). These are long-established benchmark standards for determining the involvement of GlyRs. We have also shown that this effect is insensitive to Mg^{2+} block, thus confirming a lack of NMDA receptor involvement. Hence, given the above complications, we believe that the diagnostic tests we have used are sufficient and indeed they have been accepted as such for other articles recently published in the Journal of Physiology (e.g. Werynska et al., 2023; Ankri et al., 2024).

References:

Ankri, L., Riccitelli, S. and Rivlin-Etzion, M. (2024). A new role for excitation in the retinal direction-selective circuit. *J. Physiol*. Nov;602(22):6301-6328. doi: 10.1113/JP286581.

Chan, C.Y., Sun, H.S., Shah, S.M., Agovic, M.S., Ho, I., Friedman, E., Banerjee, S.P. (2013). Direct interaction of taurine with the NMDA glutamate receptor subtype via multiple mechanisms. *Adv. Exp. Med. Biol*., 775:45-52. doi: 10.1007/978-1-4614-6130-2_4.

Suárez, L.M. and Solís, J.M. (2006). Taurine potentiates presynaptic NMDA receptors in hippocampal Schaffer collateral axons. *Eur. J. Neurosci*. Jul;24(2):405-18. doi: 10.1111/j.1460-9568.2006.04911.x.

Werynska, K., Neumann, E., Cramer, T., Ganley, R.P., Gingras, J. and Zeilhofer, H.U. (2023). A phospho-deficient α3 glycine receptor mutation alters synaptic glycine and GABA release in mouse spinal dorsal horn neurons. *J. Physiol*. Sep;601(18):4121-4133. doi: 10.1113/JP284589.

Minor points:

Line 103: "...IPSC effects were largely independent of TTX". Do you mean insensitive towards TTX? Yes – now corrected.

Line 284 (legend to figure 1): "E)" instead of "D)". Now corrected.

Lines 612 and 613: "The picture of NMDA receptor signalling has changed considerably over in recent years...". Omit "over". Now corrected.

Lines 734 to 744: The last paragraph is too speculative and can be omitted.

Removed as requested by the reviewer.

Dear Dr Moss,

Re: JP-RP-2026-287456R1 "NMDA and Glycine receptors provide dual presynaptic regulation on climbing fibre inputs to cerebellar Purkinje cells." by Guy WJ Moss, David CH Benton, Alan D. Robertson, Matthew Caldwell, Yuiry Pankratov, Lema Imam, Trevor G. Smart, and Gareth Morris

Thank you for submitting your manuscript to The Journal of Physiology. It has been assessed by a Reviewing Editor and by 2 expert referees and we are pleased to tell you that it is acceptable for publication following satisfactory revision.

The referee reports are copied at the end of this email.

Please address all the points raised and incorporate all requested revisions or explain in your Response to Referees why a change has not been made. We hope you will find the comments helpful and that you will be able to return your revised manuscript within 4 weeks. If you require longer than this, please contact journal staff: jp@physoc.org.

Your revised manuscript should be submitted online using the link in your Author Tasks Link Not Available. This link is accessible via your account as Corresponding Author; it is not available to your co-authors. If this presents a problem, please contact journal staff (jp@physoc.org). Image files from the previous version are retained on the system. Please ensure you replace or remove any files that are being revised.

If you do not wish to submit a revised version of your manuscript, you must inform our journal staff (jp@physoc.org) or reply to this email to request withdrawal. Please note that a manuscript must be formally withdrawn from the peer review process at one journal before it may be submitted to another journal.

TRANSPARENT PEER REVIEW POLICY: To improve the transparency of its peer review process The Journal of Physiology publishes online, as supporting information, the peer review history of all articles accepted for publication. Readers will have access to decision letters, including Editors' comments and referee reports, for each version of the manuscript, as well as any author responses to peer review comments. Referees can decide whether or not they wish to be named on the peer review history document.

ABSTRACT FIGURES: Authors are expected to use The Journal's premium BioRender account to create/redraw their Abstract Figures. Information on how to access this account is here: https://physoc.onlinelibrary.wiley.com/journal/14697793/biorender-access.

This will enable Authors to create and download high-resolution figures. If authors have used the free BioRender service, they can use the instructions provided in the link above to download a high-resolution version suitable for publication.

The link provided should only be used for the purposes of this submission. Authors will be charged for figures created on this account if they are not related to this manuscript submission.

LANGUAGE EDITING AND SUPPORT FOR PUBLICATION: If you would like help with English language editing, or other article preparation support, Wiley Editing Services offers expert help, including English Language Editing, as well as translation, manuscript formatting, and figure formatting at www.wileyauthors.com/eeo/preparation. You can also find resources for Preparing Your Article for general guidance about writing and preparing your manuscript at www.wileyauthors.com/eeo/prepresources.

RIGOUR AND REPRODUCIBILITY: We recommend authors consult The Journal's Rigour and Reproducibility requirements to ensure that their manuscript is suitable for publication: https://physoc.onlinelibrary.wiley.com/pb-assets/hub-assets/physoc/documents/TJP-Rigour-and-Reproducibility-Requirements.pdf

REVISION CHECKLIST:

Check that your Methods section conforms to journal policy: https://jp.msubmit.net/cgi-bin/main.plex?form_type=display_requirements#methods

Check that data presented conforms to the statistics policy: https://jp.msubmit.net/cgi-bin/main.plex?form_type=display_requirements#statistics

Upload a full Response to Referees file. To create your 'Response to Referees' copy all the reports, including any comments from the Senior and Reviewing Editors, into a Microsoft Word, or similar, file and respond to each point, using font or background colour to distinguish comments and responses and upload as the required file type.

Please upload two versions of your manuscript text: one with all relevant changes highlighted and one clean version with no changes tracked. The manuscript file should include all tables and figure legends, but each figure/graph should be uploaded as separate, high-resolution files. The journal is now integrated with Wiley's Image Checking service. For further details, see: https://www.wiley.com/en-us/network/publishing/research-publishing/trending-stories/upholding-image-integrity-wileys-

image-screening-service

You may also upload:

- 'Potential Cover Art' for consideration as the issue's cover image
- Appropriate Supporting Information (Video, audio or data set: see https://jp.msubmit.net/cgi-bin/main.plex?form_type=display_requirements#supp).

We look forward to receiving your revised submission.

If you have any queries, please reply to this email and we will be pleased to advise.

Yours sincerely,

Katalin Toth
Senior Editor
The Journal of Physiology

REQUIRED ITEMS

- You must start the Methods section with a paragraph headed Ethical approval (https://jp.msubmit.net/cgi-bin/main.plex?form_type=display_requirements#methods).

Research must comply with The Journal's policies regarding animal experiments (https://physoc.onlinelibrary.wiley.com/hub/animal-experiments) and adherence to these policies must be stated in the manuscript.

Authors should confirm in their Methods section that their experiments were carried out according to the guidelines laid down by their institution's animal welfare committee, including an ethics approval reference number. The Methods section must contain a statement about access to food, water and housing, details of the anaesthetic regime: anaesthetic used, dose and route of administration, and method of killing the experimental animals.

EDITOR COMMENTS

Reviewing Editor:

The authors have done a good job of responding to the authors previous critiques. It would be good if the authors can add add their response about glycine to reviewer#2 comment C in the Discussion section to provide context on their conclusion.

REFEREE COMMENTS

Referee #1:

This manuscript is a resubmission responding to prior reviews, that describes the presence and function of NMDA and glycine receptors on synaptic inputs to acutely isolated Purkinje neurons. The work provides compelling evidence that these are present and points to ways they might more readily be found on other terminals.

The authors have answered most of my criticisms and clarified points quite nicely.

The findings are interesting, should be applicable to a wide audience and provides original research findings that are relevant to how cerebellar circuits function. The design is robust and the conclusions noteworthy.

My only remaining question would be whether in the intact tissue - shown for glycine application in fig 9 - the large events are also blocked by TTX.

Referee #2:

The authors have taken most of my comments into account and adapted the manuscript accordingly. The only issue that remains unresolved is the characterization of GlyRs; my report proposed the use of more agonists/antagonists than just glycine itself plus the not entirely selective antagonist strychnine. Unfortunately, the authors refused to do so, and therefore the receptor characterization remains incomplete and thus a bit speculative. Thereby, the authors missed a chance to strengthen the impact of their paper.

END OF COMMENTS

Reply.

We appreciate the comments provided again by the referees. Our replies, next to the specific points raised by the reviewers, are given in below.

Reviewing Editor:

The authors have done a good job of responding to the authors previous critiques. It would be good if the authors can add their response about glycine to reviewer#2 comment C in the Discussion section to provide context on their conclusion.

We now discuss the possible benefit of using cyanotriphenylborate (CTB) in our revised manuscript.

REFEREE COMMENTS

Referee #1:

This manuscript is a resubmission responding to prior reviews, that describes the presence and function of NMDA and glycine receptors on synaptic inputs to acutely isolated Purkinje neurons. The work provides compelling evidence that these are present and points to ways they might more readily be found on other terminals.

The authors have answered most of my criticisms and clarified points quite nicely.

The findings are interesting, should be applicable to a wide audience and provides original research findings that are relevant to how cerebellar circuits function. The design is robust and the conclusions noteworthy.

My only remaining question would be whether in the intact tissue - shown for glycine application in fig 9 - the large events are also blocked by TTX.

We did not do this experiment, but this was done in the Kawa paper that we cite. TTX blocked the glycine-stimulation of EPSCs. At the time this was interpreted as evidence that the events must arise from distant cells generating action potentials, but that is ruled out in our preparation.

Referee #2:

The authors have taken most of my comments into account and adapted the manuscript accordingly. The only issue that remains unresolved is the characterization of GlyRs; my report proposed the use of more agonists/antagonists than just glycine itself plus the not entirely selective antagonist strychnine. Unfortunately, the authors refused to do so, and therefore the receptor characterization remains incomplete and thus a bit speculative. Thereby, the authors missed a chance to strengthen the impact of their paper.

We now discuss the possible benefit of using cyanotriphenylborate (CTB) in our revised manuscript.

Dear Associate Professor Moss,

Re: JP-RP-2026-287456R2 "NMDA and Glycine receptors provide dual presynaptic regulation on climbing fibre inputs to cerebellar Purkinje cells." by Guy WJ Moss, David CH Benton, Alan D. Robertson, Matthew Caldwell, Yuiry Pankratov, Lema Imam, Trevor G. Smart, and Gareth Morris

We are pleased to tell you that your paper has been accepted for publication in The Journal of Physiology.
- The Methods section must contain details of the anaesthetic regime: anaesthetic used, dose and route of administration.

The last Word (or similar) version of the manuscript provided will be used by the Production Editor to prepare your proof. When this is ready you will receive an email containing a link to Wiley's Online Proofing System. The proof should be thoroughly checked and corrected as promptly as possible.

Authors should note that it is too late at this point to offer corrections prior to proofreading. Major corrections at proof stage, such as changes to figures, will be referred to the Editors for approval before they can be incorporated. Only minor changes, such as to style and consistency, should be made at proof stage. Changes that need to be made after proof stage will usually require a formal correction notice.

All queries at proof stage should be sent to: TJP@wiley.com.

Following copyediting and typesetting the accepted version will be published online.

Yours sincerely,

Katalin Toth
Senior Editor
The Journal of Physiology

IMPORTANT POINTS TO NOTE FOLLOWING ACCEPTANCE OF YOUR PAPER:

• IMPORTANT NOTICE ABOUT OPEN ACCESS: To assist authors whose funding agencies mandate immediate public access to published research findings, The Journal of Physiology allows authors to pay an Open Access (OA) fee to have their papers made freely available immediately on publication.

The Corresponding Author will receive an email from Wiley with details on how to register or log in to Wiley Authors where you will be able to place an order.

• You can check if your funder or institution has a Wiley Open Access Account here:
https://authors.wiley.com/author-resources/Journal-Authors/open-access/author-compliance-tool.html

• TRANSPARENT PEER REVIEW POLICY: To improve the transparency of its peer review process, The Journal of Physiology publishes online as supporting information the peer review history of all articles accepted for publication. Readers will have access to decision letters, including Editors' comments and referee reports, for each version of the manuscript as well as any author responses to peer review comments. Referees can decide whether or not they wish to be named on the peer review history document.

• You can help your research get the attention it deserves! Check out Wiley's free Promotion Guide for best-practice recommendations for promoting your work at: www.wileyauthors.com/eeo/guide. You can learn more about Wiley Editing Services which offers professional video, design, and writing services to create shareable video abstracts, infographics, conference posters, lay summaries, and research news stories for your research at: www.wileyauthors.com/eeo/promotion.

• If you would like to receive our 'Research Roundup', a monthly newsletter highlighting the cutting-edge research published in The Physiological Society's family of journals (The Journal of Physiology, Experimental Physiology, Physiological Reports, The Journal of Nutritional Physiology and The Journal of Precision Medicine: Health and Disease), please click this link, fill in your name and email address and select 'Research Roundup':
https://www.physoc.org/journals-and-media/membernews

EDITOR COMMENTS

Reviewing Editor:

The authors have done an excellent job of responding to the final critiques.
